# Presynaptic Spike Timing-Dependent Long-Term Depression in the Mouse Hippocampus

**DOI:** 10.1093/cercor/bhw172

**Published:** 2016-07-25

**Authors:** Yuniesky Andrade-Talavera, Paloma Duque-Feria, Ole Paulsen, Antonio Rodríguez-Moreno

**Affiliations:** 1 Department of Physiology, Anatomy and Cell Biology, Universidad Pablo de Olavide, ES-41013 Seville, Spain; 2 Department of Physiology, Development and Neuroscience, University of Cambridge, Cambridge CB2 3EG, UK

**Keywords:** hippocampus, NMDA receptor, spike timing-dependent plasticity, t-LTD, t-LTP

## Abstract

Spike timing-dependent plasticity (STDP) is a Hebbian learning rule important for synaptic refinement during development and for learning and memory in the adult. Given the importance of the hippocampus in memory, surprisingly little is known about the mechanisms and functions of hippocampal STDP. In the present work, we investigated the requirements for induction of hippocampal spike timing-dependent long-term potentiation (t-LTP) and spike timing-dependent long-term depression (t-LTD) and the mechanisms of these 2 forms of plasticity at CA3-CA1 synapses in young (P12–P18) mouse hippocampus. We found that both t-LTP and t-LTD can be induced at hippocampal CA3-CA1 synapses by pairing presynaptic activity with single postsynaptic action potentials at low stimulation frequency (0.2 Hz). Both t-LTP and t-LTD require NMDA-type glutamate receptors for their induction, but the location and properties of these receptors are different: While t-LTP requires postsynaptic ionotropic NMDA receptor function, t-LTD does not, and whereas t-LTP is blocked by antagonists at GluN2A and GluN2B subunit-containing NMDA receptors, t-LTD is blocked by GluN2C or GluN2D subunit-preferring NMDA receptor antagonists. Both t-LTP and t-LTD require postsynaptic Ca^2+^ for their induction. Induction of t-LTD also requires metabotropic glutamate receptor activation, phospholipase C activation, postsynaptic IP_3_ receptor-mediated Ca^2+^ release from internal stores, postsynaptic endocannabinoid (eCB) synthesis, activation of CB1 receptors and astrocytic signaling, possibly via release of the gliotransmitter d-serine. We furthermore found that presynaptic calcineurin is required for t-LTD induction. t-LTD is expressed presynaptically as indicated by fluctuation analysis, paired-pulse ratio, and rate of use-dependent depression of postsynaptic NMDA receptor currents by MK801. The results show that CA3-CA1 synapses display both NMDA receptor-dependent t-LTP and t-LTD during development and identify a presynaptic form of hippocampal t-LTD similar to that previously described at neocortical synapses during development.

## Introduction

One of the most interesting properties of the mammalian brain is its ability to change in response to experience. This property was termed plasticity by the Spanish neuroscientist Santiago Ramón y Cajal more than a century ago (Cajal 1894). Plasticity is involved in the organization of cortical maps during development, and in learning and memory processes in the adult (for review, see Malenka and Bear 2004). The most extensively studied forms of plasticity are long-term potentiation (LTP) and long-term depression (LTD) of synaptic transmission. Spike timing-dependent plasticity (STDP) is a Hebbian form of long-term synaptic plasticity found in all species studied, from insects to humans, and is a strong candidate for a synaptic mechanism underlying circuit remodeling during development as well as learning and memory (Feldman and Brecht 2005; Dan and Poo 2006; Caporale and Dan 2008; Feldman 2012). In STDP, the order and millisecond-precision relative timing of pre- and postsynaptic action potentials (spikes) determine the direction and magnitude of synaptic change. Thus, timing-dependent LTP (t-LTP) occurs when a presynaptic spike is followed by a postsynaptic spike within 10–15 ms, whereas timing-dependent LTD (t-LTD) is induced when this order is reversed (Markram et al. 1997; Bi and Poo 1998; Debanne et al. 1998; for detailed reviews of STDP, see Caporale and Dan 2008; Feldman 2012). t-LTP and t-LTD have been observed in neocortical slices using different stimulation frequencies from 0.1 to 20 Hz, indicating that this form of plasticity can be elicited at low frequencies of stimulation (Sjöström et al. 2003; Bender et al. 2006; Nevian and Sakmann 2006; Rodríguez-Moreno and Paulsen 2008). STDP has been described in hippocampal primary dissociated cultures (Bi and Poo 1998), hippocampal organotypic slice cultures (Debanne et al. 1998), and acute hippocampal slices from young animals (Meredith et al. 2003; Campanac and Debanne 2008; Kwag and Paulsen 2009). However, whereas the mechanisms of STDP have been extensively studied at neocortical synapses (Sjöström et al. 2003; Bender et al. 2006; Rodríguez-Moreno and Paulsen 2008; Rodríguez-Moreno et al. 2011, 2013), comparatively less is known about STDP at hippocampal synapses.

Conventional single-spike STDP in the hippocampus has been reported in juvenile animals (Campanac and Debanne 2008; Kwag and Paulsen 2009); with maturation, induction of t-LTP appears to require the pairing of Schaffer collateral stimulation with bursts of postsynaptic action potentials (Pike et al 1999; Meredith et al. 2003; Wittenberg and Wang 2006; Buchanan and Mellor 2007; Carlisle et al. 2008). Whereas hippocampal LTP and LTD induced by high-frequency stimulation (HFS) and low-frequency stimulation (LFS), respectively, have been studied in detail (see Bliss and Collingridge 1993; Malenka and Bear 2004; and references therein), surprisingly few studies have investigated the mechanisms involved in hippocampal t-LTP and t-LTD.

To better understand the mechanisms of plasticity during development, in the present work, we studied the properties and mechanisms of t-LTP and t-LTD induction at Schaffer collateral-CA1 synapses of young (P12–P18) mouse hippocampus using whole-cell patch-clamp recordings. P12–P18 is a critical period of brain development during which much of refinement of synaptic connections occurs. We found that NMDA receptor-dependent t-LTP and t-LTD can be reliably induced by 100 pairings of presynaptic activity with single postsynaptic spikes at 0.2 Hz. However, while t-LTP requires postsynaptic GluN2A and GluN2B subunit-containing NMDA receptors, t-LTD does not, but instead requires mGlu5 receptors, CB1 receptors, and nonpostsynaptic NMDA receptors. Furthermore, we found that presynaptic calcineurin is involved in t-LTD induction and that t-LTD is presynaptically expressed as indicated by fluctuation analysis, paired-pulse ratios (PPRs) and rate of use-dependent block of NMDA receptor currents by MK-801. Whereas 2 distinct forms of LFS-induced LTD have been described, requiring either mGlu receptor (mGluR) or postsynaptic NMDA receptor signaling (Oliet et al. 1997), our results demonstrate a new form of presynaptically expressed hippocampal t-LTD requiring postsynaptic Ca^2+^, astrocytic signaling, and nonpostsynaptic NMDA receptors for its induction, properties shared with t-LTD at neocortical synapses during development.

## Materials and Methods

### Ethical Approval

All animal procedures were in accordance with the European Union Directive 2010/63/EU on the protection of animals used for scientific purposes and were approved by the local Ethical Committees. For the experiments performed in the United Kingdom, all animal procedures were in accordance with the UK Animals (Scientific Procedures) Act 1986. C57BL/6 mice were obtained from Harlan Laboratories (Spain) and from Harlan Laboratories UK Ltd (Bicester, UK). For most experiments, C57BL/6 mouse pups of either sex at postnatal day (P) 12–18 were used, avoiding the first 7–10 postnatal days when LTP depends on cAMP-dependent protein kinase rather than Ca^2+^/calmodulin-dependent protein kinase II (Yasuda et al. 2003). A total of 133 mouse pups were used. For the study of the developmental profile of t-LTD, an additional 12 male mice between P18 and P28 were used.

### Slice Preparation

Mice were anesthetized with isoflurane (2%) and decapitated for slice preparation. Hippocampal slices were prepared as described previously (Rodríguez-Moreno et al 1998; Kohl et al. 2011). Briefly, after decapitation, the whole brain containing the 2 hippocampi was removed into ice-cold solution containing (in mM): NaCl, 126; KCl, 3; NaH_2_PO_4_, 1.25; MgSO_4_, 2; CaCl_2_, 2; NaHCO_3_, 26 and glucose, 10 (pH 7.2, 300 mOsm L^−1^), positioned on the stage of a vibrating blade microtome (Leica VT1000S) and cut coronally to obtain transverse hippocampal slices (350 µm thick). The slices were maintained continuously oxygenated (95% O_2_/5% CO_2_) in this solution for at least 1 h before use. All experiments were carried out at room temperature (22–25 °C). During experiments, slices were continuously superfused with the solution described above.

### Electrophysiological Recordings

**Figure 1.**
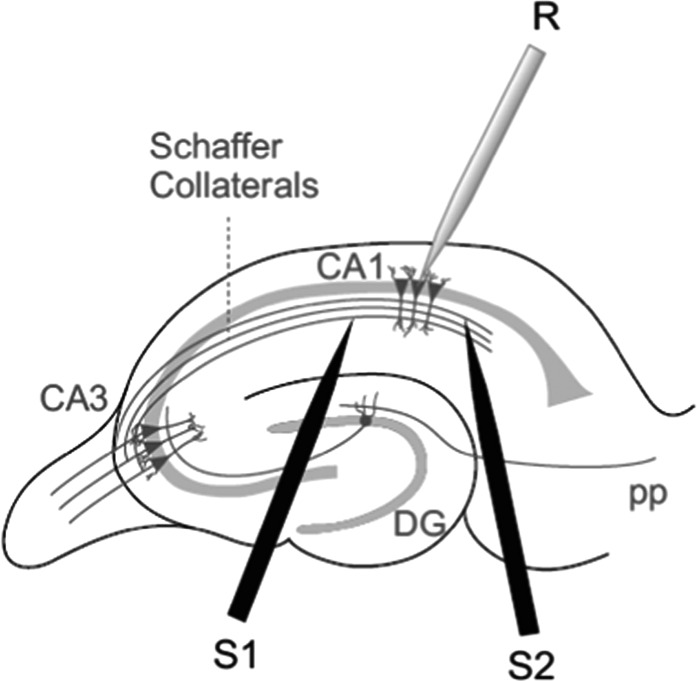
Scheme showing general experimental setup. R, recording electrode; S1 and S2, stimulating electrodes.

Whole-cell patch-clamp recordings were made from pyramidal cells located in the CA1 field of the hippocampus. CA1 pyramidal cells were patched under visual guidance by infrared differential interference contrast microscopy and verified to be pyramidal neurons by their characteristic voltage response to a current step protocol. Neurons were recorded in either voltage- or current-clamp configuration with a patch-clamp amplifier (Multiclamp 700B), and data were acquired using pCLAMP 10.2 software (Molecular Devices) or custom-made procedures in Igor Pro (WaveMetrics). Patch electrodes were pulled from borosilicate glass tubing, and had resistances of 4–7 MΩ when filled with (in mM): potassium gluconate, 110; HEPES, 40; NaCl, 4; ATP-Mg, 4; and GTP, 0.3 (pH 7.2–7.3, 290 mOsm L^−1^). Only cells with a stable resting membrane potential negative to −60 mV were included, and cells were excluded from analysis if the series resistance changed by more than 15% during the recording. All recordings were low-pass filtered at 3 kHz and acquired at 10 kHz. For plasticity experiments, EPSPs were evoked alternately in 2 input pathways, test and control, each at 0.2 Hz, by 2 monopolar stimulation electrodes placed in the “stratum radiatum” (see Fig. 1) using brief current pulses (200 µs, 0.1–0.2 mA). Stimulation was adjusted to obtain an EPSP peak amplitude of approximately 5 mV during control conditions. Pathway independence was assured by lack of cross-facilitation when the pathways were stimulated alternately with a 50 ms interval. Plasticity was assessed as changes in the slope of the EPSP, measured on its rising phase as a linear fit between time points corresponding to 25–30% and 70–75% of the peak amplitude during control conditions.

### Plasticity Protocols

After a stable EPSP baseline period of 10 min, the test input was paired 100 times with a single postsynaptic spike. The single postsynaptic spike was evoked by a brief somatic current pulse (5 ms, 0.1–0.5 pA). The control pathway was not stimulated during the pairing period. To induce t-LTD, the postsynaptic action potential was evoked within 18 ms before the onset of the EPSP, whereas, to induce t-LTP, the postsynaptic action potential was evoked within 10 ms after the onset of the EPSP. Both EPSP slopes and peak amplitudes were monitored for at least 30 min after each pairing episode. Presynaptic stimulation frequency remained constant throughout the experiment. Interleaved control t-LTP and t-LTD experiments were performed for each pharmacological compound tested.

### Pharmacology

Pharmacological agents were purchased from: Sigma-Aldrich: Fluoroacetate, thapsigargin, BAPTA, bicuculline methbromide, Tricine, Zinc chloride, d-serine, and GDPβS; Tocris Bioscience: nimodipine, (+)-MK-801 maleate, d-AP5, NBQX, TTX citrate, PPDA, Ro 25-6981 maleate, MCPG, MPEP, LY341495, AM251, 2-AG, and FK506; and Abcam: UBP-141. Salts used for internal and external solutions were purchased from Sigma-Aldrich. Compounds were dissolved in H_2_O or Ringer solution with the exception of thapsigargin, nimodipine, PPDA, FK506, THL, AM251, and 2-AG, which were dissolved in DMSO. Vehicle (DMSO) at the concentrations used did not affect baseline EPSP amplitudes and had no other detectable effects on the neurons. When investigating the effect of pharmacological agents on plasticity, all drugs were included in the superfusion fluid or patch pipette from the start of the experiment until completion (from 0 to 50 min in a standard plasticity experiment), except for fluoroacetate, which was applied from 60 min before the start of recording. When determining the effect of a pharmacological agent on baseline condition, a stable baseline of at least 10 min was first recorded and then the drug was bath applied by switching to a different perfusion line.

### Data Analysis

Data were analyzed using the Clampfit 10.2 software (Molecular Devices) and custom-made procedures in Igor Pro. The last 5 min of recording were used to estimate changes in synaptic efficacy compared with baseline. For PPR experiments, 2 EPSPs were evoked 40 ms apart for 30 s at baseline frequency at the beginning of the baseline recording and again 30 min after the end of the pairing protocol. The PPR was expressed as the slope of the second EPSP divided by the slope of the first EPSP. In experiments in which the effect of MK-801 on NMDA-EPSCs was evaluated, the half-life was estimated from a single exponential fit for each individual cell as the number of pulses required until the NMDA-EPSC peak amplitude was reduced to 50% of the baseline amplitude. Statistical comparisons were made using paired or unpaired Student's *t*-test as appropriate. *P*-values <0.05 were considered significant. Data are presented as mean ± SEM. Coefficient of variation (CV) analysis was done on EPSP slopes as previously described (Rodríguez-Moreno and Paulsen 2008).

## Results

### t-LTP and t-LTD can be Induced by Pairing Presynaptic Activity with Single Postsynaptic Action Potentials at Low Frequency in the Mouse Hippocampus

**Figure 2.**
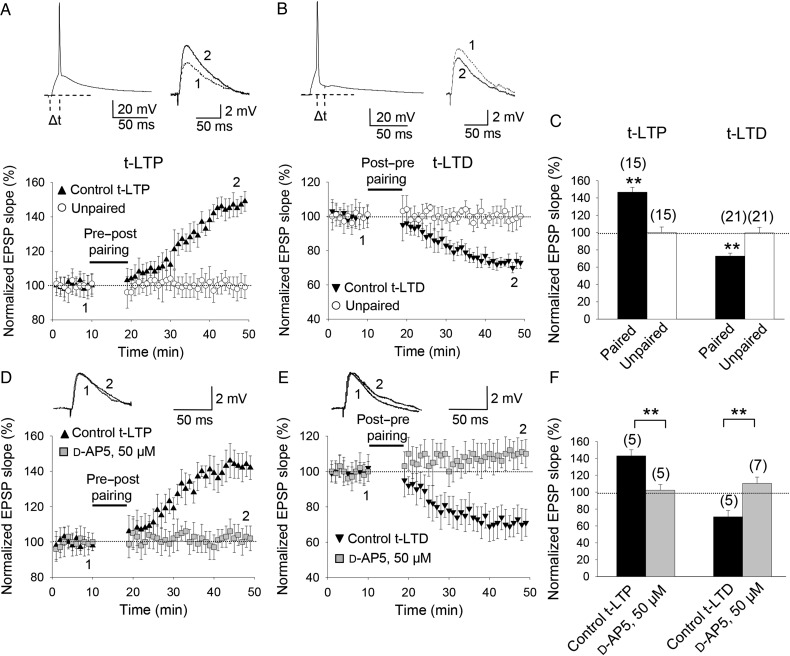
Input-specific STDP in the CA1 region of the hippocampus. (*A*) Pre-before-post, single-spike pairing protocol induced t-LTP. The EPSP slopes monitored in paired (black symbols) and unpaired pathway (open symbols) are shown. Inset, pairing protocol (Δ*t*, time between EPSP onset and peak of spike). Traces show EPSP before (1) and 30 min after (2) pairing. Potentiation was observed only in the paired pathway. (*B*) Post-before-pre, single-spike pairing protocol induced t-LTD. Symbols and traces as in (*A*). t-LTD was observed only in the paired pathway. (*C*) Summary of results. (*D*,*E*) t-LTP and t-LTD required NMDA receptors. In the presence of 50 µM d-AP5, t-LTP (*D*) and t-LTD (*E*) were completely blocked. Symbols and traces as in (*A*). (*F*) Summary of results. Error bars are SEM. **P < 0.01, unpaired Student's *t*-test. The numbers of slices are shown in parentheses.

First, we wanted to confirm that pairing presynaptic stimulation with single postsynaptic spikes at low frequency (0.2 Hz) is sufficient to induce both t-LTP and t-LTD at CA3-CA1 synapses. We monitored excitatory postsynaptic potentials (EPSPs) evoked by extracellular stimulation in the stratum radiatum during whole-cell recording of CA1 pyramidal cells in slices prepared from the mouse hippocampus (postnatal days 12–18) as previously described (Meredith et al. 2003; Kwag and Paulsen 2012). t-LTP and t-LTD were induced in current clamp using 100 pairings of single EPSPs and single postsynaptic spikes at 0.2 Hz. A pre-before-post pairing protocol (with a postsynaptic spike occurring within 10 ms of EPSP onset) elicited robust t-LTP (147 ± 6%, *n* = 15), while an unpaired control pathway was unchanged (101 ± 6%, *n* = 15; Fig. 2*A*,*C*). Conversely, a post-before-pre pairing protocol (with a postsynaptic spike occurring ∼18 ms before presynaptic stimulation) induced robust t-LTD (73 ± 4%, *n* = 21), while an unpaired control pathway remained unchanged (99 ± 6%, *n* = 21; Fig. 2*B*,*C*).

### Both t-LTP and t-LTD Require NMDA Receptors

In slices treated with the NMDA receptor antagonist d-2-amino-5-phosphonopentanoic acid (d-AP5), a pre-before-post pairing protocol failed to induce t-LTP (102 ± 7%, *n* = 5; vs. interleaved controls, 143 ± 7%, *n* = 5; Fig. 2*D*,*F*). d-AP5 also blocked t-LTD; in d-AP5-treated slices, a post-before-pre pairing protocol did not induce t-LTD (110 ± 7%, *n* = 7 vs. interleaved controls, 71 ± 8%, *n* = 5; Fig. 2*E*,*F*). These results indicate that both t-LTP and t-LTD require NMDA receptors.

### t-LTP Requires Postsynaptic NMDA Receptors Whereas t-LTD Does Not

**Figure 3.**
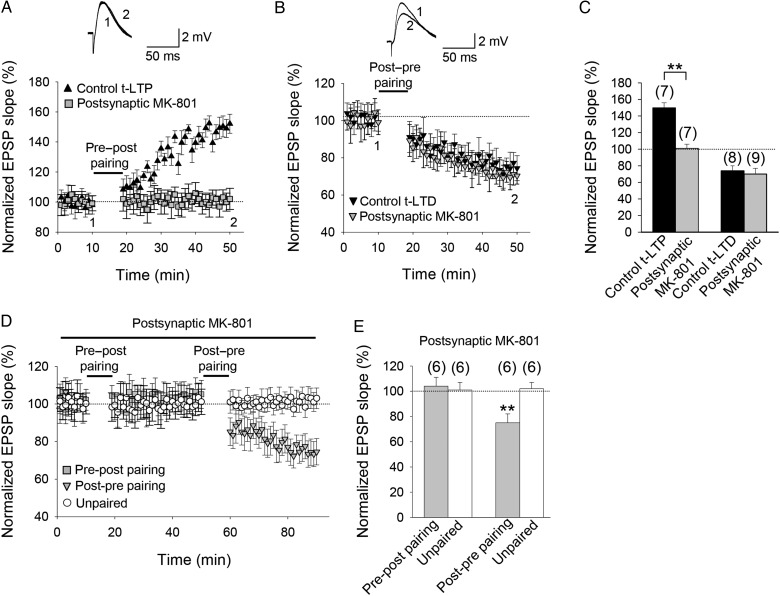
t-LTP but not t-LTD requires postsynaptic ionotropic NMDA receptors. (*A*) Postsynaptic MK-801 completely blocked induction of t-LTP. EPSP slope monitored in MK-801-treated (gray symbols) and nontreated cells (black symbols). Inset, Traces show EPSP before (1) and 30 min after (2) pairing. (*B*) Inclusion of MK-801 in the postsynaptic pipette did not block t-LTD. Symbols and traces as in (*A*). (*C*) Summary of results. (*D*) EPSP slope monitored over time in MK-801-treated neurons in a test pathway (gray symbols) and an unpaired control pathway (open symbols). After 10 min of baseline recording with 1 mM MK801 in the postsynaptic recording pipette, a pre-before-post pairing protocol in the test pathway failed to induce t-LTP and the unpaired pathway remained unchanged. Thirty minutes after the pre-before-post pairing protocol, a post-before-pre pairing protocol was applied to the same pathway. Input-specific t-LTD was induced. (*E*) Summary of results. Error bars are SEM. **P < 0.01, unpaired Student's *t*-test. The numbers of slices are shown in parentheses.

To investigate whether the NMDA receptors that are required for t-LTP and t-LTD are located postsynaptically, we repeated the pairing experiments following loading of the use-dependent NMDA receptor channel blocker MK-801 into the postsynaptic neuron via the recording patch pipette. Consistent with previous reports at neocortical synapses (Sjöström et al. 2003; Bender et al. 2006; Nevian and Sakmann 2006; Rodríguez-Moreno and Paulsen 2008), inhibiting postsynaptic NMDA receptors by including MK-801 (1 mM) in the recording pipette blocked the induction of t-LTP (101 ± 6%, *n* = 7; vs. interleaved controls, 150 ± 6%, *n* = 7; Fig. 3*A*,*C*). In contrast, t-LTD was unaffected (74 ± 6%, *n* = 8; vs. interleaved controls, 70 ± 6%, *n* = 9; Fig. 3*B*,*C*). To rule out any lack of effect due to insufficient MK-801 concentration, we repeated the experiment with 4 mM MK-801 in the recording pipette; at this concentration, as with 1 mM MK-801, t-LTP was completely prevented but t-LTD was unaffected (76 ± 6%, *n* = 5, vs. control t-LTD in interleaved slices 71 ± 7%, *n* = 5), supporting the suggestion that postsynaptic ionotropic NMDA receptors are required for t-LTP but not for t-LTD induction. To further support this conclusion, we did both pre-before-post and post-before-pre, single-spike pairing in the same cells treated with MK-801 (1 mM). Potentiation was not observed after pre-before-post pairing (104 ± 7%, *n* = 6 with an unpaired pathway unchanged, 101 ± 7%, *n* = 6; Fig. 3*D*,*E*), but subsequent post-before-pre pairing in the same pathway induced robust t-LTD (75 ± 7%, *n* = 6), while the unpaired pathway remained unchanged (102 ± 5%, *n* = 6; Fig. 3*D*,*E*). Thus, during inhibition of postsynaptic ionotropic NMDA receptors sufficient to completely block the induction of t-LTP, t-LTD could still be successfully induced.

LFS-induced NMDA receptor-dependent hippocampal LTD has been suggested to be independent of ion flow through NMDA receptors (Nabavi et al. 2013; but see Babiec et al. 2014). To exclude the possibility that the lack of effect of postsynaptic MK-801 on t-LTD is due to postsynaptic metabotropic NMDA receptor function, we investigated the effect of extracellular application of MK-801 on the induction of t-LTD. We found that 100 µM MK-801 in the superfusate completely blocked the induction of t-LTD (96 ± 8%, *n* = 6), indicating that nonpostsynaptic ionotropic NMDA receptor function is required for the induction of t-LTD.

### NMDA Receptor Subunit Dependence of t-LTP and t-LTD at CA3-CA1 Synapses of the Mouse Hippocampus

After confirming that both t-LTP and t-LTD require ionotropic NMDA receptor function, but at different locations, we wanted to determine whether this was reflected in different NMDA receptor subunit involvement.

#### t-LTP Depends on GluN2A and GluN2B Subunit-Containing NMDA Receptors

**Figure 4.**
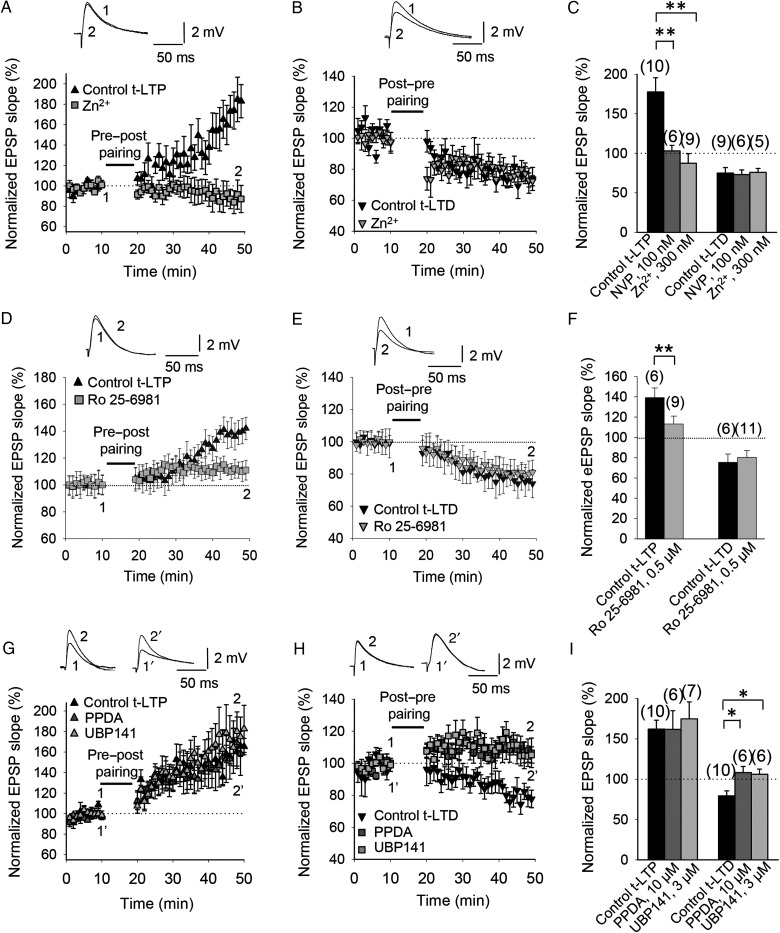
Subunit composition of NMDA receptors involved in t-LTP and t-LTD at CA3-CA1 synapses of the hippocampus. (*A*) GluN2A subunit dependence of t-LTP. t-LTP induction following a pre-before-post pairing paradigm was completely blocked by bath application of 300 nM Zn^2+^ (gray squares). (*B*) t-LTD following post-before-pre pairing was unaffected by bath application of 300 nM Zn^2+^ (gray triangles). Insets, Traces show EPSP before (1) and 30 min after (2) pairing in (*A*,*B*). (*C*) Summary of results. NVP, NVP-AAM077. (*D*) GluN2B subunit dependence of t-LTP. t-LTP induction was almost completely prevented by bath application of 0.5 µM Ro 25-6981 (gray squares). (*E*) t-LTD was unaffected by bath application of 0.5 µM Ro 25-6981 (gray triangles). Insets, EPSP before (1) and 30 min after (2) the pairing protocol in (*D*,*E*). (*F*) Summary of results. (*G*) Neither PPDA (10 µM) nor UBP-141 (3 µM) prevented t-LTP induction following a pre-before-post pairing protocol (gray triangles). (*H*) GluN2C/2D subunit dependence of t-LTD. PPDA (10 µM) blocked t-LTD following a post-before-pre pairing protocol. A more selective GluN2C/2D blocker, UBP-141 (3 µM), also blocked t-LTD (gray squares). Insets, EPSP before (1 and 1′) and 30 min after (2 and 2′) the pairing protocol. (*I*) Summary of results. Error bars are SEM. *P < 0.05, **P < 0.01, unpaired Student's *t*-test. The numbers of slices are shown in parentheses.

To test whether t-LTP and t-LTD are dependent upon GluN2A subunit-containing receptors, we used the GluN2A subunit-preferring antagonists Zn^2+^ (Bidoret et al. 2009) and NVP-AAM077 (Auberson et al. 2002). Both Zn^2+^ (300 nM) and NVP-AAM077 (100 nM) completely blocked the induction of t-LTP in P12–P18 mice (slope, 86 ± 12%, *n* = 9 and 103 ± 7%, *n* = 6, for Zn^2+^ and NVP-AAM077, respectively, vs. control slices, pooled, 177 ± 18%, *n* = 10; Fig. 4*A*,*C*), whereas t-LTD was unaffected by bath application of Zn^2+^ (76 ± 5%, *n* = 5) or NVP-AAM077 (73 ± 6%, *n* = 6) compared with interleaved control slices (75 ± 7%, *n* = 9; Fig. 4*B*,*C*). Thus, Zn^2+^ and NVP-AAM077 dissociated the NMDA receptor subunit requirement of plasticity at CA3-CA1 synapses. To further characterize the subunit composition of the NMDA receptors involved in t-LTP and t-LTD, we next investigated whether GluN2B subunit-containing NMDA receptors are necessary for the induction of t-LTP and t-LTD using the GluN2B subunit-selective noncompetitive antagonist Ro 25-6981 (Fischer et al. 1997; Banerjee et al. 2009, see Rodríguez-Moreno et al. 2010). Ro 25-6981 (0.5 µM) almost completely blocked t-LTP induction (110 ± 10%, *n* = 9) versus interleaved control slices (139 ± 8%, *n* = 6; Fig. 4*D*,*F*), but did not significantly affect t-LTD induction (80 ± 7%, *n* = 11) versus interleaved control slices (75 ± 8%, *n* = 6; Fig. 4*E*,*F*), indicating that GluN2B subunit-containing NMDA receptors are required for t-LTP but are not obligatory for t-LTD.

#### t-LTD Depends on GluN2C/2D Subunit-Containing NMDA Receptors

We next investigated the possible involvement of GluN2C/2D subunits, which are expressed during development (Monyer et al. 1994). The GluN2C/2D subunits are expressed postnatally in the hippocampus, and this expression peaks around the first week of postnatal development and then decays (Monyer et al. 1994). To test whether GluN2C/2D subunits are involved in timing-dependent plasticity at CA3-CA1 synapses in the mouse hippocampus, we used PPDA, a moderately selective, competitive antagonist at GluN2C/2D subunit-containing NMDA receptors (Morley et al. 2005). Bath application of PPDA (10 µM) did not affect t-LTP induction (161 ± 23%, *n* = 6 vs. 162 ± 11%, *n* = 10 in interleaved control slices; Fig. 4*G*,*I*), but completely blocked t-LTD (108 ± 8%, *n* = 6 vs. interleaved control slices 76 ± 6%, *n* = 10; Fig. 4*G*,*H*). A less potent but more selective antagonist, UBP-141, also selectively blocked t-LTD (106 ± 6%, *n* = 6 vs. interleaved control slices 76 ± 6%, *n* = 10; Fig. 4*H*,*I*) with no effect on t-LTP (175 ± 21%, *n* = 7, vs. interleaved control slices, 162 ± 11%, *n* = 10; Fig. 4*G*,*I*). These results indicate that t-LTP requires GluN2A and GluN2B, but not GluN2C/2D subunit-containing NMDA receptors, whereas t-LTD requires NMDA receptors that contain GluN2C and/or GluN2D subunits.

### t-LTD Requires Postsynaptic Ca^2+^

**Figure 5.**
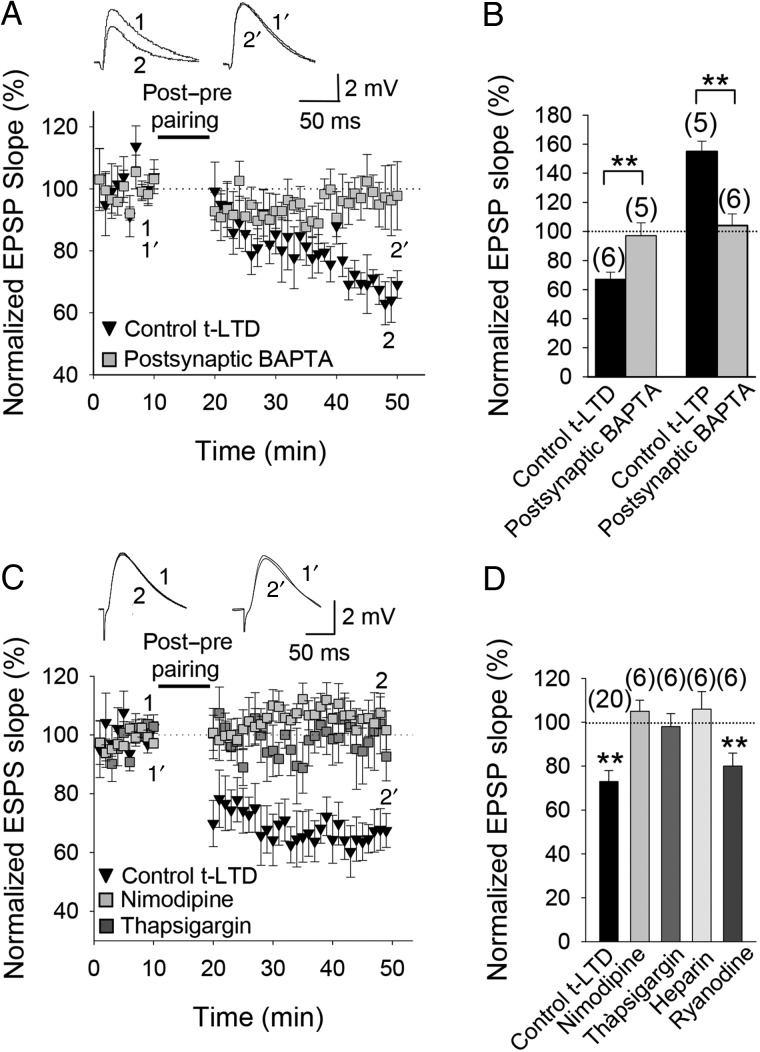
Calcium sources for t-LTD. (*A*) t-LTD was prevented by BAPTA (20 mM) in the postsynaptic recording pipette. Insets show EPSP before (1 and 1′) and after (2 and 2′) post-before-pre pairing in control conditions and with BAPTA in the postsynaptic pipette. (*B*) Summary of results. Both t-LTD and t-LTP were blocked by postsynaptic BAPTA. (*C*) Nimodipine or blocking Ca^2+^ release from internal stores with thapsigargin (10 µM) prevented t-LTD. The EPSP slopes monitored in paired control slices (black symbols) and in paired slices treated with nimodipine (gray symbols) or thapsigargin (dark gray symbols). Traces show EPSP before (1 and 1′) and 30 min after (2 and 2′) pairing in slices treated with nimodipine (1 and 2) or thapsigargin (1′ and 2′). (*D*) Summary of results. Nimodipine, thapsigargin and heparin (400 U/mL) all blocked induction of t-LTD, shown versus the pooled interleaved controls (73 ± 8%, *n* = 18), whereas ryanodine did not. Error bars are SEM. **Indicates *P* < 0.01, unpaired Student's *t*-test. The numbers of slices are shown in parentheses.

While t-LTD seems not to require postsynaptic ionotropic NMDA receptors, both t-LTP and t-LTD have been shown to require postsynaptic Ca^2+^ at neocortical synapses (Bender et al. 2006; Nevian and Sakmann 2006; Rodríguez-Moreno et al. 2013). We therefore investigated the postsynaptic Ca^2+^ requirement of t-LTD at hippocampal CA3-CA1 synapses by loading the Ca^2+^ chelator BAPTA into the postsynaptic cell via the patch pipette. t-LTD was prevented when BAPTA (20 mM) was included in the recording pipette (97 ± 9%, *n* = 5, vs. interleaved controls, 67 ± 5%, *n* = 6) as was t-LTP (104 ± 8%, *n* = 6, vs. interleaved controls, 155 ± 7%, *n* = 5; Fig. 5*A*,*B*), indicating that both t-LTP and t-LTD require postsynaptic Ca^2+^. If t-LTD does require postsynaptic Ca^2+^ but NMDA receptors are not the source of this postsynaptic Ca^2+^, what is the source and function of this postsynaptic Ca^2+^ required for the induction of t-LTD? It has previously been reported that Ca^2+^ channels and release of Ca^2+^ from intracellular stores are required for t-LTD induction at neocortical synapses (Bender et al. 2006; Nevian and Sakmann 2006). To investigate this possibility at the CA3-CA1 synapses of the hippocampus, we first repeated the pairing protocols following bath application of an L-type Ca^2+^ channel blocker, nimodipine; in the presence of nimodipine (10 µM), t-LTD induction was completely prevented (105 ± 7%, *n* = 6, vs. interleaved controls, 75 ± 9%, *n* = 5; Fig. 5*C*,*D*). Next, we performed the t-LTD pairing protocol in the presence of thapsigargin, which depletes intracellular Ca^2+^ stores; in the presence of bath-applied thapsigargin (10 µM), t-LTD was also prevented (98 ± 6%, *n* = 6 vs. interleaved controls, 65 ± 6%, *n* = 5; Fig. 5*C*,*D*). The presence of heparin (400 U/mL), a blocker of IP_3_R-mediated Ca^2+^ release (Ghosh et al. 1988; Khodakhah and Armstrong 1997), in the recording pipette also completely prevented t-LTD induction (106 ± 8%, *n* = 6 vs. interleaved control slices, 73 ± 8%, *n* = 5; Fig. 5*D*), suggesting that postsynaptic IP_3_R-mediated Ca^2+^ release is required for t-LTD. In contrast, inclusion in the patch pipette of ryanodine (100 µM), a blocker of ryanodine receptors (RyRs) and Ca^2+^-induced Ca^2+^ release from internal stores, did not prevent induction of t-LTD (80 ± 8%, *n* = 6 vs. 72 ± 8% in interleaved control slices, *n* = 5, Fig. 5*D*), suggesting that IP_3_R-mediated Ca^2+^ release, but not RyR-sensitive Ca^2+^ stores, is required for t-LTD..

### t-LTD Requires Activation of mGluRs

**Figure 6.**
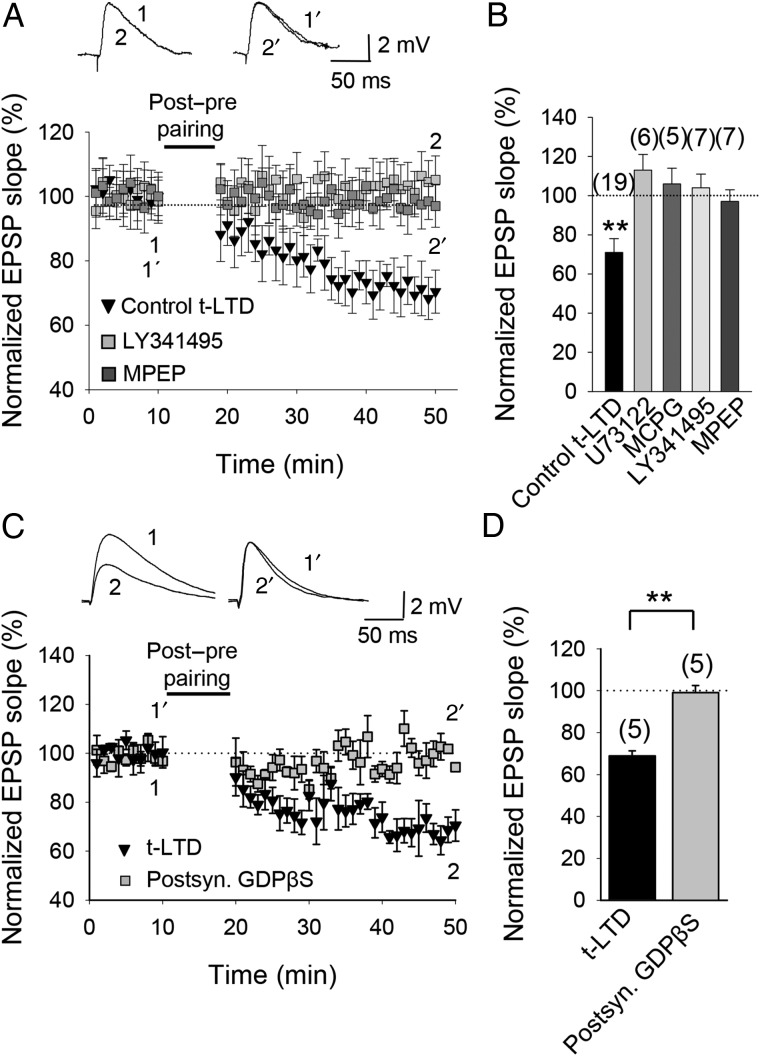
Metabotropic glutamate receptor involvement in t-LTD. (*A*) t-LTD requires mGlu5 receptors and PLC signaling. The EPSP slopes monitored in control slices (black symbols) and in slices treated with the mGluR antagonist LY341495 (gray symbols) or the mGlu5 receptor antagonist MPEP (dark gray symbols) following post-before-pre pairing. Inset, Traces show EPSP before (1 and 1′) and 30 min after (2 and 2′) pairing in slices treated with LY341495 (1 and 2) and in slices treated with MPEP (1′ and 2′). (*B*) Summary of results. (*C*) t-LTD requires activation of postsynaptic metabotropic receptors. Time course of t-LTD induction in control conditions (black symbols) and with the postsynaptic neuron loaded with GDPβS. Inset, Traces show EPSP before (1 and 1′) and 30 min after pairing (2 and 2′) in control slices (1 and 2) and with the postsynaptic neuron loaded with GDPβS (1′ and 2′). (*D*) Summary of results. Error bars are SEM. **P < 0.01, unpaired Student's *t*-test. The numbers of slices are shown in parentheses.

The source of IP_3_ responsible for Ca^2+^ release from intracellular stores during induction of t-LTD might be through activation of phospholipase C (PLC). We tested this possibility by using the PLC inhibitor U73122. t-LTD was completely prevented by bath application of U733122 (10 µM, 113 ± 8%, *n* = 6; Fig. 6*B*), confirming the involvement of the PLC pathway in t-LTD. The activation of PLC during the induction protocol could occur through the activation of mGluRs; in fact, in neocortical as well as in hippocampal neurons the induction of some types of t-LTD has been reported to require the stimulation of mGluRs (Otani and Connor 1998; Anwyl 1999; Egger et al. 1999), which activates PLC to produce IP_3_ (Berridge 1998). We therefore tested whether t-LTD at CA3-CA1 synapses requires mGluRs. In the presence of the broad-spectrum mGluR antagonists MCPG (500 µM) or LY367385 (100 µM), t-LTD was completely prevented (MCPG, 106 ± 8%, *n* = 5; LY341495, 104 ± 7%, *n* = 7; Fig. 6*A*,*B*). Neither MCPG nor LY367385 affected baseline EPSP slope (not shown). t-LTD was also blocked by the specific mGlu5 receptor antagonist MPEP (20–40 µM, 97 ± 6%, *n* = 7, vs. interleaved control slices for the 3 experimental conditions, pooled together, 70 ± 8%, *n* = 19; Fig. 6*A*,*B*). Neither MCPG nor LY367385 prevented t-LTP induction (MCPG, 153 ± 8%, *n* = 5; LY367385, 155 ± 6%, *n* = 5). These results suggest that t-LTD requires an mGlu5 receptor-mediated increase of intracellular Ca^2+^ from intracellular stores. To test the possible postsynaptic location of the metabotropic receptors involved in t-LTD we repeated the experiments with the postsynaptic neuron loaded with GDPβS to prevent G-protein-mediated signaling. In this condition, t-LTD was completely prevented (99 ± 5%, *n* = 5 vs. interleaved control slices with no GDPβS loaded into postsynaptic cells 69 ± 4%, *n* = 5, Fig. 6*C*,*D*). These results indicate that postsynaptic G protein-coupled receptors, possibly mGluRs, are involved in t-LTD..

### t-LTD Requires Endocannabinoid Signaling

**Figure 7.**
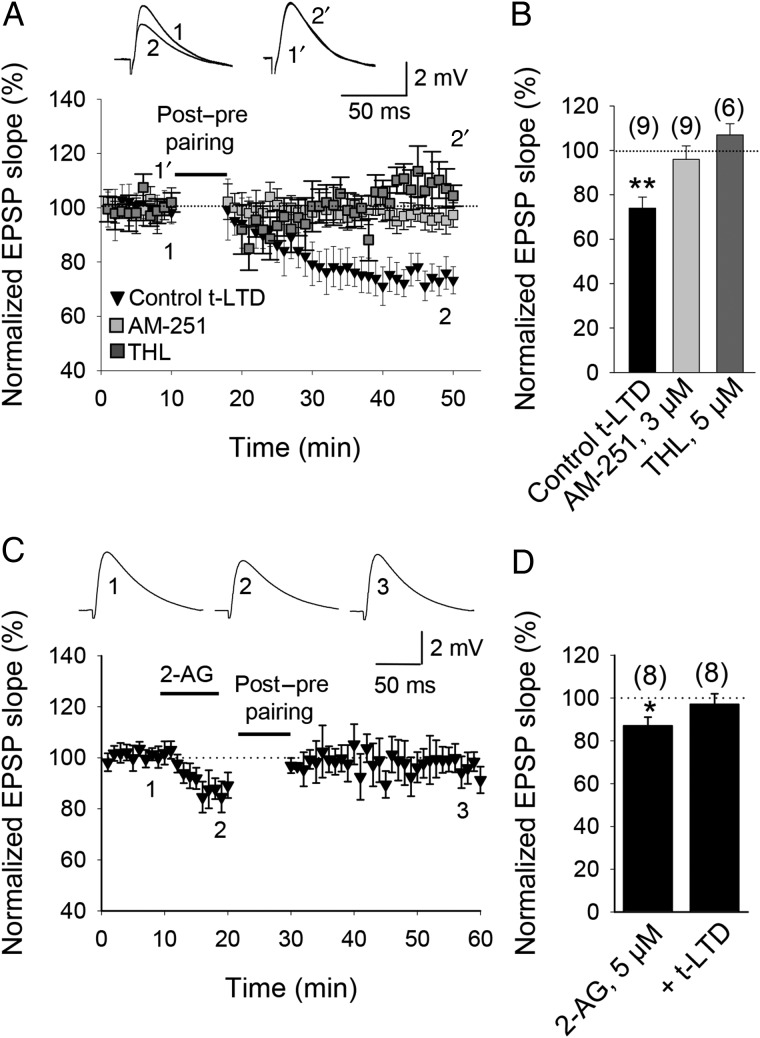
Cannabinoid receptor involvement in t-LTD. (*A*) t-LTD requires activation of CB1 receptors. Time course of t-LTD induction in control conditions (black symbols) and in slices treated with the CB1 receptor antagonist AM251 following post-before-pre pairing. Inset: Traces show EPSP before (1 and 1′) and 30 min after pairing (2 and 2′) in control slices (1 and 2) and in slices treated with AM251 (1′ and 2′). (*B*) Summary of results. Note that in the presence of THL, t-LTD was completely prevented. (*C*) 2-AG effect on EPSPs. After 2-AG t-LTD induction is prevented. Inset: Traces show baseline EPSP (1), after 2-AG (2), and 30 min after pairing (3). (*D*) Summary of results. Error bars are SEM. *P < 0.05, **P < 0.01, unpaired Student's *t*-test. The numbers of slices are shown in parentheses.

Endocannabinoids (eCBs) are synthetized and released by postsynaptic cells in response to depolarization, Ca^2+^ elevation and/or mGluR signaling, and some synapses require signaling by eCBs for plasticity (Auclair et al. 2000; Gerdeman et al. 2002; Marsicano et al. 2002; Robbe et al. 2002; Chevaliere and Castillo 2003; Huang et al. 2003; Sjöström et al. 2003; Safo and Regehr 2005; Bender et al. 2006; Nevian and Sakmann 2006; Perea and Araque 2007; Navarrete and Araque 2008; Min and Nevian 2012; Gómez-Gonzalo et al. 2015). To investigate whether t-LTD at CA3-CA1 synapses of the mouse hippocampus requires postsynaptic eCB synthesis, we performed t-LTD experiments with the postsynaptic neuron loaded via the patch pipette with tetrahydrolipstatin (THL, 5 µM), an inhibitor of the eCB synthesizing enzyme diacylglycerol lipase. In this experimental condition, t-LTD induction was completely prevented (107 ± 5%, *n* = 6, vs. interleaved control slices, 66 ± 9%, *n* = 5; Fig. 7*A*,*B*), thus indicating that postsynaptic eCB synthesis is necessary for t-LTD. eCBs diffuse and activate CB1 receptors on presynaptic neurons and/or glial cells (Sjöström et al. 2003; Navarrete and Araque 2010; Min and Nevian 2012). To investigate the involvement of CB1 receptors in hippocampal t-LTD, we repeated the t-LTD induction protocol in the presence of the CB1 receptor antagonist AM251 (3 µM). In the presence of AM251, t-LTD was completely prevented (96 ± 6%, *n* = 9, vs. interleaved slices, 74 ± 5%, *n* = 5; Fig. 7*A*,*B*). To investigate whether CB1 receptors are necessary only during t-LTD induction or also during expression, we applied AM251 after the t-LTD induction protocol. AM251 applied 20 min after the t-LTD induction protocol did not affect the magnitude of t-LTD (49 ± 17%, *n* = 4 vs. 57 ± 16% in interleaved control slices, Supplementary Fig. 1). Thus, t-LTD induction requires activation of postsynaptic eCB synthesis and activation of CB1 receptors. THL prevents the formation of the eCB 2-arachidonylglycerol (2-AG). To more directly investigate the possible involvement of 2-AG during t-LTD induction, we bath-applied 2-AG. A small reduction in the slope of the EPSP was observed (87 ± 5%, *n* = 8, Fig. 7*C*,*D*). Importantly, following 2-AG application, a t-LTD pairing protocol was no longer able to induce t-LTD (97 ± 6, *n* = 8, Fig. 7*C*,*D*), suggesting that 2-AG occludes the induction of t-LTD and that 2-AG or a structurally similar eCB is a component of the signaling cascade required for the induction of hippocampal t-LTD..

### t-LTD Requires Astrocyte Signaling and d-serine

**Figure 8.**
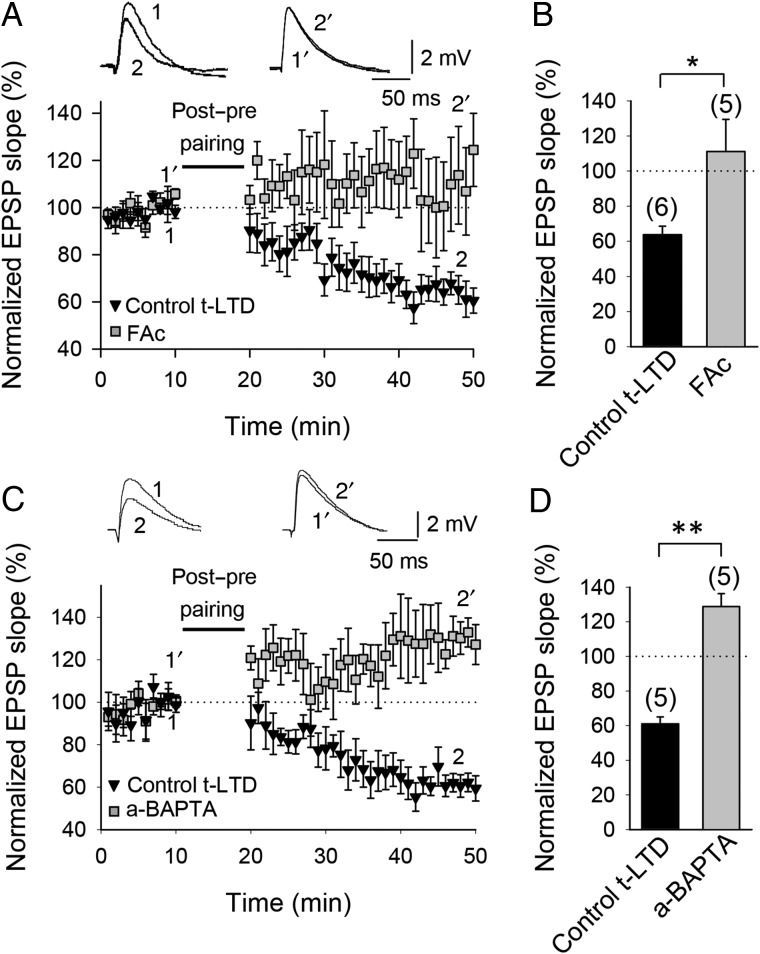
Astroglial involvement in t-LTD. (*A*) Time course of t-LTD induction in control conditions (black symbols) and absence of t-LTD in fluoroacetate (FAc)-treated slices (gray symbols). Inset, Traces show EPSP before (1 and 1′) and 30 min after pairing (2 and 2′) in control slices (1 and 2) and in slices treated with FAc (1′ and 2′). (*B*) Summary of results. (*C*) Astrocyte-neuron dual recordings performed during t-LTD induction in control conditions (astrocyte loaded with the same intracellular solution as used for neurons; black symbols) and with astrocytes loaded with the calcium chelator BAPTA via the recording pipette (a-BAPTA; gray symbols). Inset: Traces show EPSP before (1 and 1′) and 30 min after pairing (2 and 2′) in control conditions (1 and 2) and in a-BAPTA conditions (1′ and 2′). (*D*) Summary of results. Error bars are SEM. *P < 0.05, **P < 0.01, unpaired Student's *t*-test. The numbers of slices are shown in parentheses.

**Figure 9.**
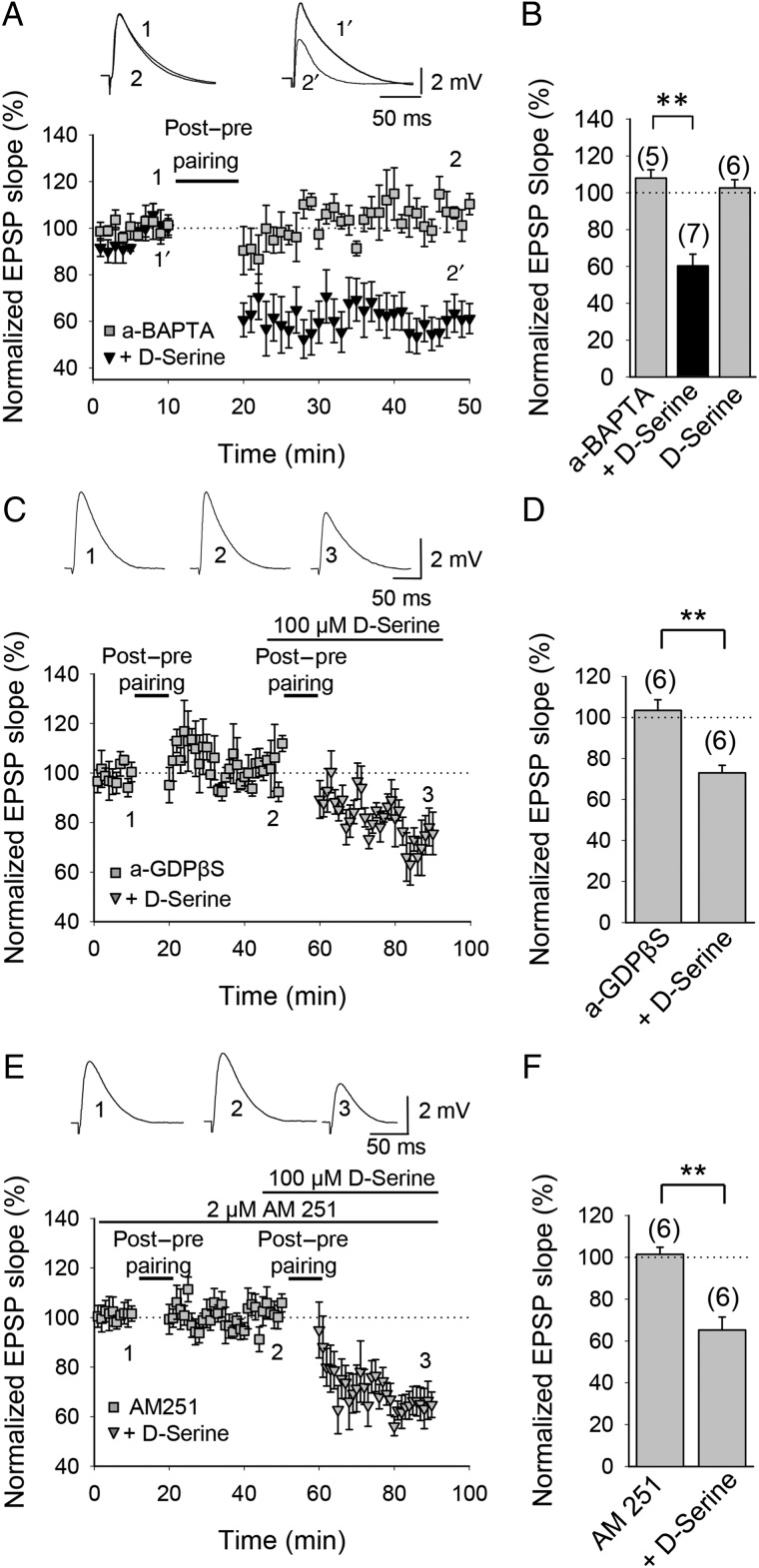
Astroglial d-serine involvement in t-LTD. (*A*) d-Serine recovers t-LTD in recordings with BAPTA-treated astrocytes. Astrocyte-neuron dual recordings during post-before-pre pairing with the calcium chelator BAPTA included in the astrocyte via the recording pipette (a-BAPTA conditions) without (gray squares) and in the presence of 100 µM d-serine (black triangles). Inset: Representative traces from baseline (1) and 30 min after pairing protocol (2) in a-BAPTA conditions and from baseline (1′) and 30 min after pairing protocol (2′) in a-BAPTA conditions in the presence of 100 µM d-serine. (*B*) Summary of results. Gray column labeled ‘d-Serine’ shows a-BAPTA condition without pairing protocol. (*C*) d-Serine recovers t-LTD in recordings with GDPβS-treated astrocytes. Astrocyte-neuron dual recordings performed during post-before-pre pairing with GDPβS included in the astrocyte via the recording pipette (a-GDPβS conditions) without (gray squares) and in the presence of 100 µM d-serine (gray triangles). Inset: Representative traces from baseline (1) and 30 min after pairing protocol (2) in a-GDPβS conditions and 30 min after applying again the t-LTD induction protocol in the presence of 100 µM d-serine (3). (*D*) Summary of results. (*E*) d-Serine recovers t-LTD in AM251-treated slices. In the presence of AM251 t-LTD induction is prevented (gray squares) and recovered in the presence of 100 µM d-serine (gray triangles). Inset: Representative traces from baseline (1) and 30 min after pairing protocol (2) in AM251-tretaed slices and 30 min after applying again the t-LTD induction protocol in the presence of 100 µM d-serine (3). (*F*) Summary of results. Error bars are SEM. **P < 0.01, unpaired Student's *t*-test. The numbers of slices are shown in parentheses.

CB1 receptors have been localized to presynaptic boutons (Llano et al. 1991; Rodríguez et al. 2001; Alger 2002; Freund et al. 2003; Chevaleyre et al. 2006) and astrocytes (Rodríguez et al. 2001; Salio et al. 2002; Stella 2004; Navarrete and Araque 2008; Min and Nevian 2012). In astrocytes, CB1 receptor activation has been suggested to stimulate the release of glutamate and other gliotransmitters, including d-serine (Araque et al. 2014). To investigate a possible involvement of astrocytes in the induction of hippocampal t-LTD, we preincubated the slices for 1 h with the gliotoxin fluoroacetate (10 mM); this completely abolished t-LTD (111 ± 18%, *n* = 5 vs. interleaved control slices, 64 ± 7%, *n* = 6; Fig. 8*A*,*B*). In contrast, t-LTP was resistant to fluoroacetate treatment, as a pre-before-post pairing protocol still induced t-LTP in fluoroacetate-treated slices (136 ± 6%, *n* = 6 vs. interleaved control slices, 141 ± 11%, *n* = 6, Supplementary Fig. 2). Next, using a patch pipette, we loaded individual astrocytes with the Ca^2+^ chelator BAPTA (20 mM) for 1–4 h before patching a pyramidal neuron. Ca^2+^-dependent release of gliotransmitters is prevented by this treatment (see Parpura and Zorec 2010 and references therein). We recorded CA1 pyramidal neurons in the proximity (50–100 µm) of the BAPTA-loaded astrocyte and found that BAPTA loading of the astrocyte (a-BAPTA) prevented the induction of t-LTD at CA3-CA1 synapses (121 ± 8%, *n* = 5 vs. interleaved control slices, 61 ± 6%, *n* = 5; Fig. 8*C*,*D*). t-LTP was not affected by BAPTA loading into nearby astrocytes as a pre-before-post pairing protocol still induced t-LTP in this experimental condition (147 ± 4%, *n* = 5, vs. interleaved control experiments, 151 ± 8%, *n* = 6; Supplementary Fig. 2). These results suggest that astrocytic Ca^2+^-dependent gliotransmitter release is necessary for t-LTD induction. d-serine, a co-agonist at NMDA receptors, is a candidate gliotransmitter, and astrocytes in CA1 have been reported to release d-serine (Henneberger et al. 2010; Zhuang et al. 2010). To test whether d-serine might be responsible for the requirement of astrocytes during induction of t-LTD, we repeated the BAPTA-loading experiments in the presence of 100 µM d-serine added to the superfusion fluid. In this experimental condition, t-LTD was completely recovered (60 ± 8%, *n* = 7, vs. interleaved slices with a-BAPTA, 108 ± 4%, *n* = 5; Fig. 9*A*,*B*). d-serine without the pairing protocol did not affect baseline transmission, as the EPSP slope was not affected by application of d-serine to the superfusion fluid for 30 min without a pairing protocol (103 ± 4%, *n* = 6; Fig. 9*B*, Supplementary Fig. 3). This result supports the conclusion that the contribution of astrocytes during induction of t-LTD involves the release of a co-agonist at NMDA receptors, most likely d-serine, as t-LTD in the a-BAPTA condition but in the presence of d-serine was restored to a level similar to that of control t-LTD experiments. To investigate a possible mechanism involved in d-serine release, we repeated the experiments loading the astrocytes with the G-protein signaling blocker GDPβS and recording neurons in the proximity. In this experimental condition, t-LTD was also completely prevented (103 ± 5%, *n* = 6). In the same cells, the subsequent application of d-serine recovered t-LTD (73 ± 5%, *n* = 6, Fig. 9*C*,*D*). This result suggests that a G protein-dependent mechanism is involved in the astrocytic signaling required for t-LTD. Consistent with a role for CB1 receptors in activating astrocytes, application of 100 µM d-serine also completely rescued t-LTD in the presence of the CB1 receptor antagonist AM251 (65 ± 5%, *n* = 5, vs. 101 ± 3%, *n* = 5 in the presence of 3 µM AM251 only; Fig. 9*E*,*F*). These results suggest that CB1 receptors, possibly located on astrocytes, are controlling astrocytic release of a co-agonist at nonpostsynaptic NMDA receptors. Interestingly, and consistent with the results of Min and Nevian (2012) in the rat barrel cortex, direct stimulation of astrocytes by depolarizing current pulses paired with low-frequency presynaptic activity without postsynaptic action potentials was sufficient to induce LTD (64 ± 14%, *n* = 5, Supplementary Fig. 4), suggesting that, in this experimental condition, astrocytes may contribute to synaptic depression by mechanisms additional to that of release of a co-agonist at NMDA receptors.

.

### Presynaptic Calcineurin Involvement in t-LTD

**Figure 10.**
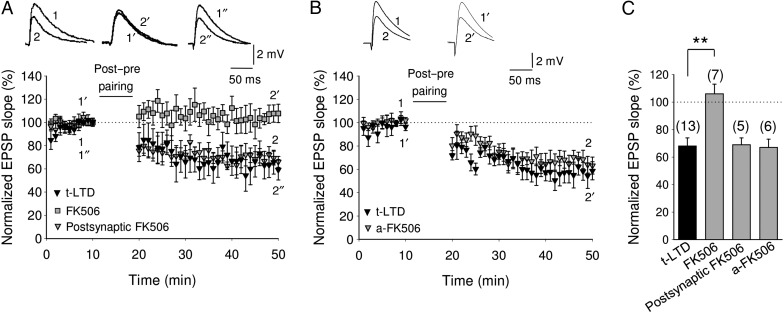
Presynaptic calcineurin is involved in t-LTD at CA3-CA1 synapses. (*A*) Time course of effect of post-before-pre pairing in control conditions (black triangles) and in FK506 (10 µM)-treated slices (bath applied, gray squares) or loaded into the postsynaptic cell via the patch pipette (gray triangles). Inset, Traces show EPSP before (1, 1′ and 1″) and 30 min after pairing (2, 2′ and 2″) in control slices (1 and 2) and in slices treated with FK506 in the bath (1′ and 2′) or loaded into the postsynaptic cell (1″ and 2″). (*B*) Time course of effect of post-before-pre pairing in control conditions (black triangles) and in FK506 (10 µM)-treated slices loaded into astrocytes via the patch pipette (gray triangles). Inset, Traces show EPSP before (1 and 1′) and 30 min after pairing (2 and 2′) in control slices (1 and 2) and in slices treated with FK506 loaded into the astrocytes (1′ and 2′). Note that t-LTD induction is prevented by adding FK506 to the bath, whereas it is not affected by loading the inhibitor into the postsynaptic cell or into the astrocyte. (*C*) Summary of results. Error bars are SEM. **P < 0.01, unpaired Student's *t*-test. The numbers of slices are shown in parentheses.

To gain mechanistic insight into how activation of nonpostsynaptic NMDA receptors could lead to t-LTD, we conjectured that a Ca^2+^-dependent enzyme might be involved. Since the Ca^2+^-dependent protein phosphatase calcineurin has earlier been implicated in other forms of LTD, both in the hippocampus (Mulkey et al. 1994) and neocortex (Torii et al. 1995; Rodríguez-Moreno et al. 2013), we tested the effect of the calcineurin inhibitor FK506 on hippocampal t-LTD. We found that while t-LTD was not affected by loading the postsynaptic neuron or an astrocyte with FK506 (10 µM) via a patch pipette (69 ± 5%, *n* = 5 and 67 ± 5%, *n* = 6, respectively; Fig. 10*A*–*C*), it was completely blocked by bath application of FK506 (10 µM) (106 ± 7%, *n* = 7 vs. 68 ± 6%, *n* = 13 in interleaved control slices; Fig. 10*A*,*C*). FK506 was active when loaded into the postsynaptic neurons as loading the same concentration of FK506 into neurons of layer 2 of the medial entorhinal cortex completely prevented LTD in synaptic input from layer 1 (not shown), as previously reported (Kourrich et al. 2008), indicating that presynaptic calcineurin is involved in the induction of hippocampal t-LTD..

### Presynaptic Expression of t-LTD

The fact that t-LTD was blocked by extracellular application, but not by postsynaptic intracellular application, of an NMDA receptor channel blocker raises the possibility that nonpostsynaptic, possibly presynaptic, NMDA receptors are necessary for t-LTD, as demonstrated at neocortical synapses (Bender et al. 2006; Nevian and Sakmann 2006; Corlew et al. 2007, 2008, Rodríguez-Moreno and Paulsen 2008).

**Figure 11.**
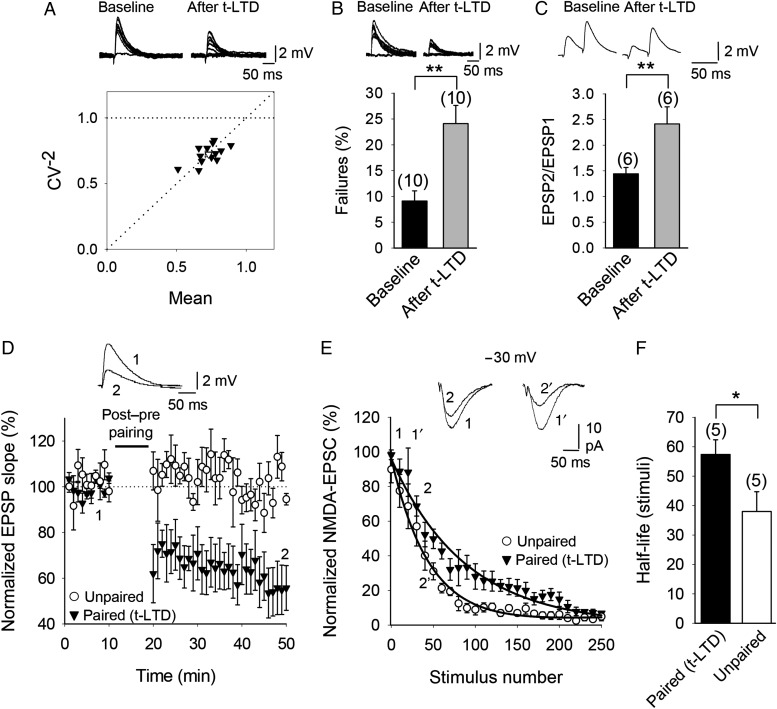
t-LTD at CA3-CA1 synapses is presynaptically expressed. (*A*) CV analysis is consistent with presynaptic expression of t-LTD. Normalized plot of CV^−2^ versus mean EPSP slope yielded points along the diagonal following induction of t-LTD. Inset, Example traces during baseline and 30 min after induction of t-LTD. (*B*) Number of failures increased after t-LTD induction. Inset, Example traces during baseline and 30 min after induction of t-LTD. (*C*) PPR increased after t-LTD. Inset, Example traces during baseline and 30 min after induction of t-LTD. (*D*) EPSP slopes monitored in paired (black triangles) and unpaired pathway (white circles). Traces show EPSP before (1) and 30 min after (2) t-LTD induction protocol in the paired pathway. Only the paired pathway showed t-LTD. (*E*) NMDA receptor-mediated EPSC peak amplitudes monitored in the same cells after bath application of MK-801 at the end of the EPSP recordings shown in (*D*). A single exponential function was fitted to the experimental data in both pathways. A slower decay of NMDAR-EPSC amplitudes was observed in the paired pathway (black triangles) compared with the unpaired pathway (white circles). (*F*) The half-life was estimated from the fitted function for each individual experiment in paired and unpaired pathways. The error bars are SEM. *P < 0.05, **P < 0.01, unpaired Student's *t*-test. The numbers of slices are shown in parentheses.

To determine the site of expression of hippocampal t-LTD, we combined several approaches. First, we estimated the noise-subtracted CV of the synaptic responses before and after t-LTD induction. A plot of CV^−2^ versus the change in the mean evoked EPSP slope (M) before and after t-LTD yielded points along the diagonal line indicating a presynaptic modification of release parameters (Malinow and Tsien 1990; Fig. 11*A*). Second, in several experiments we observed failures in synaptic transmission, and thus we analyzed whether a change in the number of failures occurred after t-LTD. A consistent increase in the number of failures after t-LTD was observed in our experiments (24 ± 4%, *n* = 10 after t-LTD vs. 9 ± 2% in baseline, *n* = 10) suggesting also a presynaptic locus of expression of this form of t-LTD (Fig. 11*B*). Third, we analyzed the PPRs during baseline and 30 min after a t-LTD pairing protocol was applied. The analysis of PPRs before and after t-LTD showed a significant increase of PPR after t-LTD (2.4 ± 0.3, *n* = 6 vs. 1.4 ± 0.1 during baseline, *n* = 6; *P* < 0.01 Student's *t*-test), which is also indicative of a presynaptic change (Fig. 11*C*). Finally, to further corroborate the presynaptic expression of t-LTD, we analyzed the progressive block of NMDA receptor-mediated currents by MK-801 after induction of t-LTD compared with a control pathway (Hessler et al. 1993; Rosenmund et al. 1993; Rodríguez-Moreno et al. 2011). As MK-801 is an irreversible NMDA receptor open-channel blocker (Jahr 1992), NMDA receptors are blocked only at synapses that release transmitter, so the trial-to-trial progressive rate of block provides a measure of the release probability (Rosenmund et al. 1993). We induced t-LTD in 1 pathway by 100 pairings of a postsynaptic action potential followed after 18 ms by an EPSP. This post-before-pre pairing protocol induced robust t-LTD (55 ± 11%, *n* = 5; Fig. 11*D*). An unpaired naïve pathway served as a control, and no change in EPSPs slope was observed in that pathway (105 ± 5%, *n* = 5; Fig. 11*D*). Thirty minutes after the pairing protocol, we switched to voltage-clamp mode and recorded NMDAR-EPSCs at −30 mV in the same cells. NMDA receptor currents were isolated by the addition of gabazine (2 µM) and NBQX (10 µM) to the superfusion solution to block GABA_A_ and AMPA/kainate receptors, respectively. Following bath application of 100 µM MK-801, a gradual decrease of the amplitude of the NMDA receptor currents, and eventually an almost complete block, was observed in the paired as well as the unpaired pathway. However, a slower rate of decay (measured as the number of stimuli necessary to reduce NMDAR-EPSC amplitude) was observed in the paired compared with the unpaired pathway (Fig. 9*E*). The half-life of the NMDAR-EPSC was 40 ± 5 stimuli for the unpaired pathway (*n* = 5) and 68 ± 4 stimuli for the paired pathway (*n* = 5; Fig. 11*F*). These results are consistent with a reduction in the neurotransmitter release probability in the paired pathway. Together, these results are all indicative of a presynaptic locus of expression of this form of t-LTD..

## Discussion

In summary, we have found here that both t-LTP and t-LTD can be induced at hippocampal CA3-CA1 synapses in young mice by pairing presynaptic activity with single postsynaptic action potentials at low stimulation frequency (0.2 Hz). These 2 forms of plasticity both require NMDA receptors for their induction, but the NMDA receptors required for t-LTP and t-LTD are at different locations; while t-LTP requires postsynaptic ionotropic NMDA receptors, t-LTD does not. We have further characterized the subunit composition of these 2 different populations of NMDA receptors and found that the postsynaptic NMDA receptors required for t-LTP contain GluN2A and GluN2B, but not GluN2C or GluN2D subunits, whereas NMDA receptors mediating t-LTD contain GluN2C and/or GluN2D subunits. Both t-LTP and t-LTD require postsynaptic Ca^2+^ for their induction, and t-LTD also requires L-type voltage-dependent Ca^2+^ channels, activation of mGlu5 receptors, PLC and postsynaptic IP_3_ receptor-mediated Ca^2+^ release from internal stores, postsynaptic eCB synthesis, activation of CB1 receptors, and astroglial signaling. We have furthermore found that application of the NMDA receptor co-agonist d-serine bypasses the requirement of astrocytes, suggesting that astrocytes deliver a co-agonist at NMDA receptors during induction of t-LTD. Finally, we demonstrated that t-LTD is presynaptically expressed as indicated by analysis of trial-to-trial EPSP fluctuations and failure rates, as well as PPRs and the rate of use-dependent depression of NMDA receptor currents by MK-801. This new form of hippocampal t-LTD is in many aspects similar to t-LTD at layer 4-to-layer 2/3 synapses in neocortex (Bender et al. 2006; Nevian and Sakmann 2006; Rodríguez-Moreno and Paulsen 2008). However, one notable difference is the nature of the putative gliotransmitter released by astrocytes, which in the case of layer 4 to layer 2/3 synapses is glutamate (Min and Nevian 2012), while our data at hippocampal CA3-CA1 synapses suggest the involvement of d-serine or another co-agonist acting on nonpostsynaptic ionotropic NMDA receptors.

### Location of NMDA Receptors in Timing-dependent Plasticity

Both t-LTP and t-LTD require NMDA receptors as both were prevented by the inclusion of the NMDA receptor antagonist d-AP5 in the superfusion fluid. Similar to neocortical layer 4-to-layer 2/3 synapses we found that while postsynaptic ionotropic NMDA receptors are required for hippocampal t-LTP, they are not required for t-LTD as the inclusion of the NMDA receptor channel blocker MK-801 (1 or 4 mM) in the postsynaptic cell prevented induction of t-LTP but not t-LTD, indicating that NMDA receptors required for t-LTP and t-LTD are at different locations. Importantly, extracellular application of MK-801 blocked the induction of t-LTD, demonstrating that nonpostsynaptic ionotropic NMDA receptor function is required for t-LTD, but an additional role for a postsynaptic NMDA receptor-mediated metabotropic effect cannot be completely ruled out (Nabavi et al. 2013).

### NMDA Receptor Subunits in Timing-dependent Plasticity

The presence of different subpopulations of NMDA receptors in different brain regions suggests that different subtypes play different roles in brain function (Cull-Candy and Leszkiewicz 2004). By using different subunit-preferring pharmacological agents, it was reported that LTP and LTD induced by high-frequency and low-frequency afferent stimulation, respectively, could be dissociated, with LTP being dependent of GluN2A, but not GluN2B subunit-containing receptors, and LTD requiring GluN2B, but not GluN2A subunit-containing receptors in the hippocampus (Liu et al. 2004) as well as in the perirhinal cortex (Massey et al. 2004). From these first studies the situation has been shown to be more complex (Berberich et al. 2005; Toyoda et al. 2005; Weitlauf et al. 2005; Morishita et al. 2007). Most of the available antagonists have limitations in selectivity and caution should be exercised when interpreting these data (Neyton and Paoletti 2006). Nevertheless, in our experiments, the GluN2A antagonists Zn^2+^ and NVP-AAM077 (at 100 nM) both completely abolished t-LTP at CA3-CA1 synapses in the hippocampus without affecting t-LTD, whereas the GluN2C and 2D subunit-preferring antagonists PPDA and UBP141 both completely blocked t-LTD without affecting t-LTP. The GluN2B subunit-selective antagonist Ro 25-6981 significantly reduced t-LTP without significantly affecting t-LTD. These results are similar to the double dissociation found at neocortical layer 4-to-layer 2/3 synapses in the sense that t-LTP was blocked by GluN2A subunit-preferring NMDA receptor antagonists but not GluR2C/2D subunit-preferring NMDA receptor antagonists and vice versa (Banerjee et al. 2009), but, whereas a GluN2B subunit-selective NMDA receptor antagonist reduced t-LTP in the hippocampus, t-LTP was not affected in the somatosensory neocortex (Banerjee et al. 2009). Nevertheless, both in the hippocampus and neocortex, the differential NMDA receptor requirement for the induction of t-LTP and t-LTD might reflect compartment-specific expression of different NMDA receptor subunits (Duguid and Sjöström 2006).

### Ca^2+^ Requirements of t-LTP and t-LTD and the Role of mGlu5 Receptors

We found that both t-LTP and t-LTD require a rise in postsynaptic Ca^2+^, as both were blocked by the presence of BAPTA in the postsynaptic cell, similar to what was reported in the somatosensory cortex (Bender et al. 2006; Nevian and Sakmann 2006). t-LTD requires Ca^2+^ from intracellular stores as Ca^2+^ store depletion (by thapsigargin) and postsynaptic IP_3_ receptor blockade (using heparin) blocked t-LTD induction. This form of t-LTD thus resembles previously described postsynaptic NMDA receptor-independent forms of LTD (Anwyl 1999; Svoboda and Mainen 1999; Nosyreva and Huber 2005), which often involves voltage-dependent Ca^2+^ channels (Oliet et al. 1997). This was also observed in our results, since t-LTD was blocked by the L-type Ca^2+^ channel blocker nimodipine. These requirements of hippocampal t-LTD are also shared with presynaptic t-LTD at neocortical synapses (Sjöström et al. 2003; Rodríguez-Moreno and Paulsen 2008). The rise in intracellular Ca^2+^ may be due to the activation of mGlu5 receptors and subsequent activation of PLC as t-LTD was prevented by the general metabotropic glutamate receptor antagonists MCPG and LY341495 as well as the selective mGlu5 receptor antagonist MPEP and intracellular loading of the PLC inhibitor U73122. Moreover, t-LTD induction was prevented when the postsynaptic neuron was loaded with GBPβS, which blocks G-protein-mediated signaling. However, presynaptic mGluRs have also been described at these synapses, modulating glutamate release (Rodríguez-Moreno et al. 1998) and participating in plasticity (Gómez-Gonzalo et al. 2015). The existence of glial mGluRs has also been reported (Porter and McCarthy 1996; Perea and Araque 2005) and they might also have a role in this form of t-LTD. Loading GDPβS into astrocytes prevented t-LTD induction, but not the synaptic depression induced by mGluR activation (Supplementary Fig. 5); thus, this result does not allow us to distinguish between the possibilities that GDPβS blocks t-LTD due to inhibition of mechanisms mediated by mGluRs or by other types of metabotropic receptor, such as CB1 receptors. More work will be necessary to clarify this point.

### Endocannabinoids and CB1 Receptors in t-LTD

Postsynaptic loading of THL, a selective inhibitor of the eCB synthesizing enzyme diacylglycerol lipase, blocked, and addition of the eCB 2-AG occluded, the induction of t-LTD, suggesting that eCBs are involved in the induction of t-LTD. Whether postsynaptic Ca^2+^ or another signaling cascade is required for the release of eCBs is not known. eCBs released from pyramidal neurons have also been reported to induce long-term enhancement of evoked glutamate release though CB1 receptor activation and stimulation of astrocytes (Gómez-Gonzalo et al. 2015). In our experiments, AM-251, a CB1 receptor antagonist, prevented the induction of t-LTD, suggesting that eCB binding to CB1 receptors is required for induction of t-LTD. CB1 receptors involved in synaptic plasticity are located in the presynaptic neuron (Sjöström et al 2003) and/or in astrocytes (Navarrete and Araque 2008, 2010; Min and Nevian 2012). In the present work, we did not elucidate the site of CB1 receptors mediating t-LTD and further work will be required to address this question, although the demonstration that astrocytes are required for t-LTD is suggestive that astrocytic CB1 receptors might be involved.

### Astrocytes are Required for Induction of t-LTD

We used 2 methods to investigate the involvement of astrocytic activity in t-LTD. First, the gliotoxin fluoroacetate prevented the induction of t-LTD. Second, loading individual astrocytes with the Ca^2+^ chelator BAPTA completely abolished t-LTD, suggesting that Ca^2+^-regulated release of a gliotransmitter is necessary for t-LTD induction at hippocampal CA3-CA1 synapses.

A role for astrocytes in synaptic depression is well established. For instance, ATP released from astrocytes as a result of neuronal activity can modulate synaptic transmission in cultured hippocampal neurons. ATP tonically suppresses glutamatergic transmission via P2Y receptors. This effect depends on the presence of co-cultured astrocytes (Zhang et al. 2003). Glutamate activates non-NMDA receptors on astrocytes and triggers ATP release which causes homo- and heterosynaptic depression (Zhang et al. 2003). GABAergic network activation of glial cells can also induce hippocampal heterosynaptic depression (Serrano et al. 2006). In addition, astrocytes have been implicated in transient heterosynaptic depression in the CA1 region of acute hippocampal slices (Andersson et al. 2007).

### d-Serine Recovers t-LTD

The NMDA receptor co-agonist d-serine is an interesting candidate for a gliotransmitter involved in t-LTD induction (Panatier et al. 2006; Duffy et al. 2008; Zhang et al. 2008). Indeed, addition of d-serine to the superfusion fluid completely restored t-LTD in experiments in which astrocytes were loaded with BAPTA, suggesting that astrocytes release a gliotransmitter required for t-LTD, acting as co-agonist on nonpostsynaptic NMDA receptors.

Additionally, d-serine recovered t-LTD when astrocytes were loaded with GDPβS and when AM251 was added in the superfusion fluid. These results are consistent with the possibility that CB1 receptors are located on the astrocytes and that d-serine acts on nonpostsynaptic NMDA receptors involved in mediating t-LTD. GluN3A subunit-containing presynaptic NMDA receptors are required for t-LTD in the mouse visual cortex during development (Larsen et al. 2011), and di-heteromeric GluN1/GluN3A NMDA receptors without glutamate-binding sites have been suggested to exist (Paoletti et al. 2013). In support of this possibility, pairing of astrocytic activity with presynaptic activity without postsynaptic action potentials was sufficient to induce LTD. However, the finding that in the presence of d-serine post-before-pre pairing, rather than only presynaptic activation, was required to induce LTD is consistent with the involvement of NMDA receptors that also contain glutamate-binding GluN2 subunits. It remains possible that astrocytes might release both glutamate and d-serine.

### Possible Presynaptic NMDA Receptors in the Hippocampus

The results obtained are suggestive that hippocampal t-LTD is mediated by presynaptic NMDA receptors (Banerjee et al. 2016), as ionotropic NMDA receptors are involved but postsynaptic ionotropic NMDA receptors are not required, as demonstrated by the failure of postsynaptic MK-801 to block t-LTD. In the somatosensory cortex, a similar form of t-LTD has been described that requires presynaptic NMDA receptors (Rodríguez-Moreno and Paulsen 2008; see also Bender et al. 2006; Nevian and Sakmann 2006; Corlew et al. 2007, 2008). While the focus of the present work was the characterization of hippocampal t-LTP and t-LTD and early insights into the underlying mechanisms, there is no definitive demonstration of the location of the NMDA receptors involved in hippocampal t-LTD. Future work using paired recordings at CA3-CA1 synapses and including MK-801 in the presynaptic neuron will determine whether the nonpostsynaptic NMDA receptors involved in hippocampal t-LTD have a presynaptic location. The intracellular uncaging of a caged form of MK-801 could more precisely identify the location of these receptors in the cells, as has been done at layer 4-to-layer 2/3 synapses in somatosensory cortex (Rodríguez-Moreno et al. 2011; Reeve et al. 2012). The presence of presynaptic NMDA receptors in the hippocampus has been suggested previously from experiments monitoring noradrenaline release in synaptosomes (Pittaluga and Raiteri 1990, 1992; Wang et al. 1992), and immuno-electron microscopy has observed NMDA receptor immunolabeling at presynaptic elements of the hippocampus (Siegel et al. 1994; Charton et al. 1999; Jourdain et al. 2007). Physiological roles for presynaptic NMDA receptors have also been suggested, including modulating transmitter release by acting as autoreceptors (Mameli et al. 2005; Jourdain et al. 2007; see Corlew et al. 2008 for review). Presynaptic NMDA receptors have been suggested to be involved in STDP in visual cortex (Sjöström et al. 2003; Corlew et al. 2007; Larsen et al. 2011, 2014) and somatosensory cortex (Bender et al. 2006; Brasier and Feldman 2008; Urban-Ciecko et al. 2014) with direct evidence obtained at layer 4-to-layer 2/3 neurons of somatosensory cortex (Rodríguez-Moreno and Paulsen 2008; Rodríguez-Moreno et al. 2011). In the hippocampus, presynaptic NMDA receptors have been suggested to participate in the induction of LTP (McGuinness et al. 2010). Future experiments should elucidate their precise location and physiological roles at hippocampal CA3-CA1 synapses.

### Calcineurin is Necessary for t-LTD Induction

Protein phosphatases, including calcineurin, have been reported to be required for several different forms of LTD, both in the hippocampus (Mulkey et al. 1994) and neocortex (Torii et al. 1995). Using FK506 to block calcineurin activity, our results indicate the involvement of presynaptic calcineurin in t-LTD induction, in similarity to the involvement of presynaptic calcineurin in neocortical p-LTD (Rodríguez-Moreno et al. 2013).

While the exact mechanisms of how calcineurin mediates LTD of evoked transmitter release are unknown, several presynaptic proteins involved in transmitter release processes are targets of phosphorylation/dephosphorylation cascades and therefore candidates to mediate such changes. These include proteins involved in exocytosis, endocytosis, and the regulation of the size of the releasable, recycling and reserve pools of synaptic vesicles (Leenders and Sheng 2005; Kim and Ryan 2010; Bykhovskaia 2011), as well as presynaptic calcium channels and their association to the release machinery (Kaeser and Südhof 2005; Catterall and Few 2008; Su et al. 2012; Kim and Ryan 2013). Future experiments should determine the exact mechanism by which calcineurin mediates LTD of evoked glutamate release.

### t-LTD is Presynaptically Expressed at Hippocampal CA3-CA1 Synapses

We used 3 different approaches to determine the locus of expression of this form of t-LTD, and all 3, fluctuation analysis (including failure rate), PPR and MK801-induced progressive decay of NMDA receptor currents were consistent with presynaptic changes, strongly suggesting that the locus of expression of this form of hippocampal t-LTD is presynaptic.

### What is the Physiological Role of This Form of Plasticity?

The exact role of STDP in the hippocampus is not known, and more work is necessary to determine specific functions for t-LTP and t-LTD. Temporally asymmetric synapse strengthening has been predicted to drive learning of sequences (Hebb 1949). Computational models have indicated that temporally asymmetric LTP in the hippocampus could store sequences of spatial positions and that place fields shift backward along repeated paths due to LTP at synapses from earlier- to later-activated place cells (Blum and Abbott 1996). Experimental support for this model was found by Mehta et al. (1997), consistent both with simple Hebbian STDP (Mehta et al. 1997) and with a unified model of rate- and timing-dependent plasticity (Yu et al. 2008). Bush et al. (2010) showed that a rate- and timing-dependent plasticity model could explain both learning of spatial sequences and increased functional connectivity between neurons with overlapping place fields. Thus, STDP seems a good candidate to mediate spatial learning and the possible role of t-LTP and t-LTD in forms of learning involving the hippocampus will be addressed in future studies.

Our studies were done in developing P12–P18 animals, and the form of t-LTD studied here was absent after P21 (Supplementary Fig. 6). The functions of t-LTP and t-LTD during development are most probably related to the refinement of synaptic connections and remodeling of neuronal circuits. As a Hebbian learning rule, t-LTP should occur when the spike order is pre-before-post, strengthening those connections in which the presynaptic neuron takes part in firing the postsynaptic cell, as predicted by Hebb, whereas t-LTD occurs when the spike order is reversed, so that noncausal spiking weakens the connections involved, possibly as a first step in the elimination of those connections during development in a similar way to that suggested in the neocortex (see Caporale and Dan 2008). The form of t-LTD described here may be specifically related to development as it was only observed until the third week of development (in fact, after P21 the same protocol induced t-LTP; Supplementary Fig. 6). Whether or not the specific form of t-LTP studied here is present in adult animals remains to be confirmed (see also Meredith et al. 2003; Wittenberg and Wang 2006).

**Figure 12.**
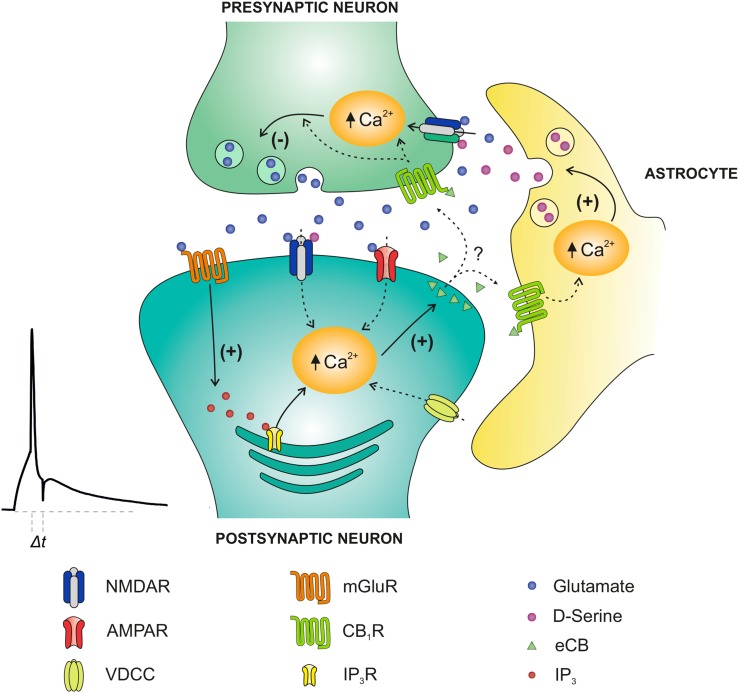
Model of presynaptic t-LTD at CA3-CA1 synapses of the hippocampus. t-LTD is induced by a post-before-pre, single-spike pairing protocol. Postsynaptic action potentials activate voltage-dependent Ca^2+^ channels (VDCCs), and presynaptically released glutamate activates postsynaptic mGlu5 receptors, which synergistically activate PLC, producing IP_3_, which causes Ca^2+^ release from internal stores, and DAG, which serves as precursor for eCBs synthesis. The eCB signal leads to activation of CB1 receptors, facilitating d-serine release from astrocytes, which, together with glutamate released from presynaptic neurons, activates presynaptic NMDA receptors on Schaffer collateral boutons. This leads to an increase in presynaptic Ca^2+^, activation of calcineurin and synaptic depression.

In summary, these results indicate that at CA3-CA1 synapses of mouse hippocampus, both t-LTP and t-LTD can be induced at low frequency by temporal pairing of presynaptic activity with single postsynaptic spikes. Both t-LTP and t-LTD require NMDA receptors but these NMDA receptors are different and with different location; whereas t-LTP requires postsynaptic NMDA receptors located at CA1 neurons, t-LTD does not require postsynaptic ionotropic NMDA receptors but requires nonpostsynaptic, likely presynaptic NMDA receptors. We also studied the mechanisms involved in t-LTD and found that its induction requires postsynaptic Ca^2+^ through L-type Ca^2+^ channels as well as mGlu5 receptor activation and release of Ca^2+^ from internal stores, which mediates the synthesis and release of eCBs activating CB1 receptors located on astrocytes and/or on presynaptic neurons. Astrocytes were shown to be crucial for the induction of t-LTD most likely by releasing the gliotransmitter d-serine, which, together with glutamate released by presynaptic neurons, activates presynaptic NMDA receptors to induce hippocampal t-LTD (Fig. 12).

## Supplementary Material

Supplementary material can be found at: http://www.cercor.oxfordjournals.org/.

## Funding

This work received support from the Ministerio de Ciencia e Innovación/European Regional Development Fund (grant BFU2009-10034), Ministerio de Economía y Competitividad/European Regional Development Fund (grant BFU2012-38208) and the Junta de Andalucía/European Regional Development Fund (grant P11-CVI-7290 to A.R.-M.), from the Biotechnology and Biological Sciences Research Council, UK (grant BB/H002383/2 to O.P.), and from a Royal Society International Exchanges grant to A.R.-M. and O.P. Funding to pay the Open Access publication charges for this article was provided by Research Councils UK.

## Supplementary Material

Supplementary Data
