# Supplementary material for: Presynaptic Spike Timing-Dependent Long-Term Depression in the Mouse Hippocampus
Source: Cereb Cortex. 2016 Jul 25;26(8):3637–54. doi: 10.1093/cercor/bhw172 (PMC4961031; doi:10.1093/cercor/bhw172)
Supplement: Supplementary Data [file supp_26_8_3637__index.html]

Presynaptic Spike Timing-Dependent Long-Term Depression in the Mouse Hippocampus — Presynaptic Spike Timing-Dependent Long-Term Depression in the Mouse Hippocampus — Supplementary Data 

# Presynaptic Spike Timing-Dependent Long-Term Depression in the Mouse Hippocampus

## Supplementary Data

Supplementary Data

- Supplementary Data - Pdf file
