# Supplementary material for: Presynaptic Spike Timing-Dependent Long-Term Depression in the Mouse Hippocampus
Source: Cereb Cortex. 2016 Jul 25;26(8):3637–54. doi: 10.1093/cercor/bhw172 (PMC4961031; doi:10.1093/cercor/bhw172)
Supplement: Supplementary Data [file supp_bhw172_bhw172supp.pdf]

Supplementary Figures

Presynaptic spike timing-dependent long-term depression in the mouse hippocampus

Andrade-Talavera, Y, Duque-Feria P, Paulsen O and Rodríguez-Moreno A

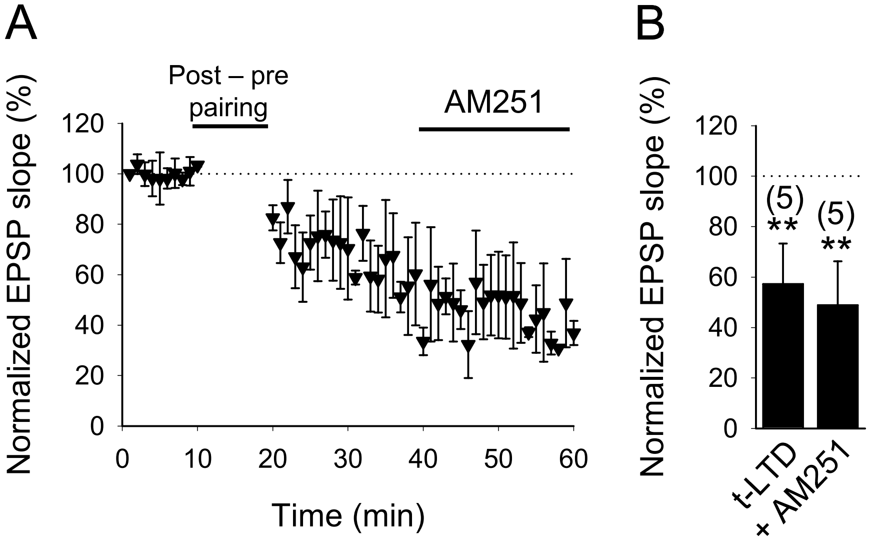

Figure S1. CB1 receptor activation is not necessary for t-LTD maintenance.

CB1 receptor antagonism with AM251 20 minutes after the pairing protocol did not affect t-LTD.

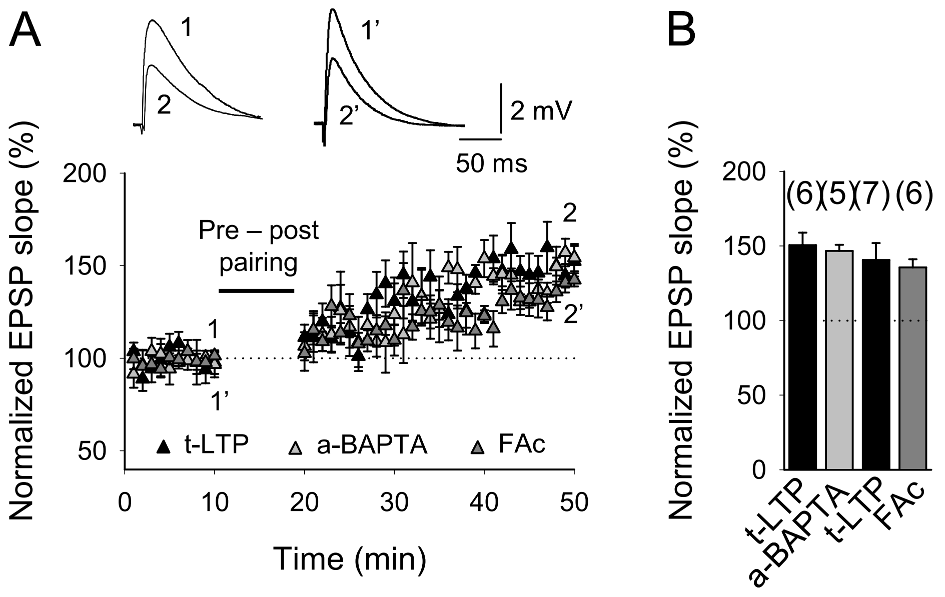

Figure S2. **t-LTP does not require astroglial signaling at CA3-CA1 synapses.**

(A) Time course of t-LTP in control conditions (black symbols), in dual recordings performed in neurons and a neighboring astrocyte with the inclusion of the calcium chelator BAPTA into the astrocyte (gray symbols) and in slices treated with the gliotoxin fluoracetate (dark gray symbols). Inset: Representative traces from baseline (1) and 30 minutes after pairing protocol (2) in control conditions and from baseline (1') and 30 minutes after pairing protocol (2') in dual recordings performed with astrocytes loaded with BAPTA. (B) Summary of results. Error bars are S.E.M. The numbers of slices are shown in parentheses.

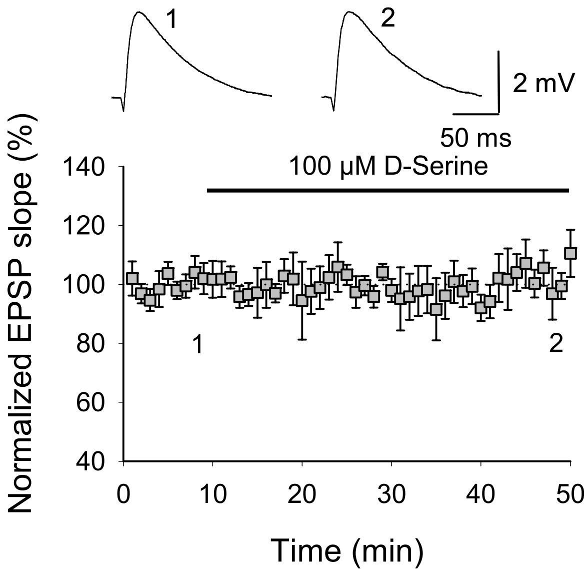

Figure S3. D-Serine does not affect basal synaptic transmission at CA3-CA1 synapses. EPSP slope monitored 10 minutes before and 40 minutes after D-serine application (baseline: 101 ± 4% D-serine: 103 ± 4%; n= 6). Traces show EPSP from baseline (1) and 40 minutes after D-serine application (2).

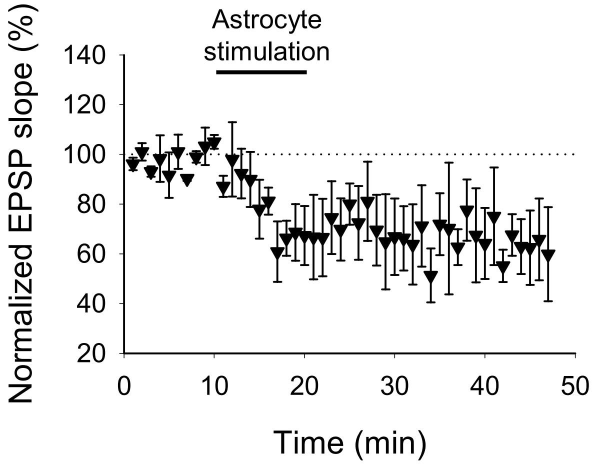

Figure S4. Astrocyte stimulation induces LTD in neighboring pyramidal neuron at CA3-CA1 hippocampal synapses. Time course of the EPSP slope monitored from dual recordings performed in astrocytes and neighboring pyramidal neurons. Astrocyte depolarization from -80 mV to 0 mV for 500 ms at 0.4 Hz, during 10 minutes while EPSP were evoked by basal stimulation at 0.2 Hz induced a robust a-LTD (64 ± 14%; n = 3).

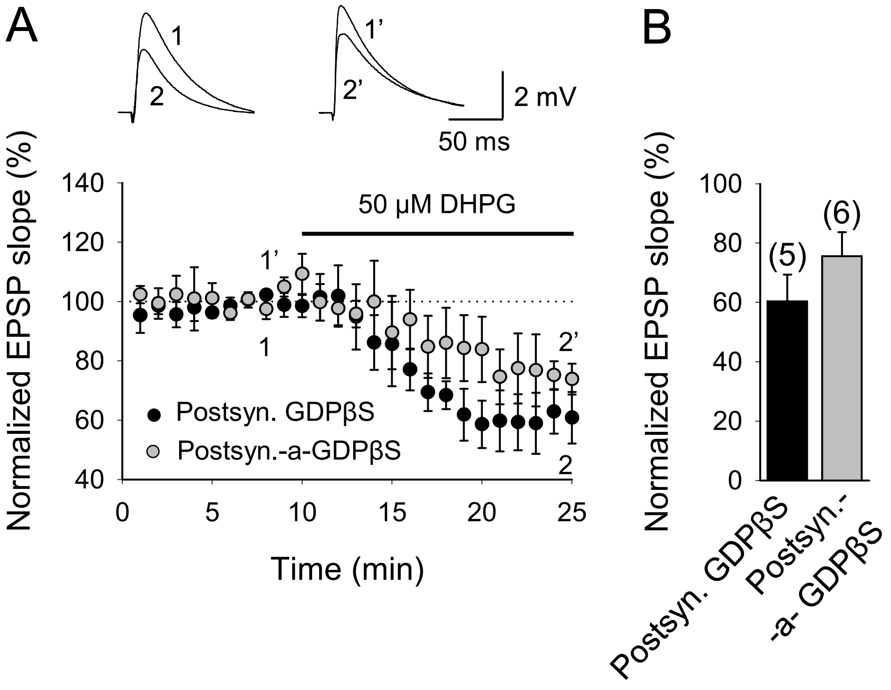

Figure S5. The activation of group I mGluR depresses synaptic transmission at CA3-CA1 synapses. (A) The group I mGluR agonist DHPG caused a depression of the EPSP slope monitored in GDPβS-filled postsynaptic neurons (black symbols) and in dual recordings when both postsynaptic neurons and astrocytes were filled with GDPβS (gray symbols). Traces show EPSPs from a GDPβ-S-filled postsynaptic neuron before (1) and after DHPG application (2) and from dual recordings in a GDPβS-filled postsynaptic neuron and astrocyte before (1') and after DHPG application (2'). (B) Summary of results. Error bars are S.E.M. The numbers of slices are shown in parentheses.

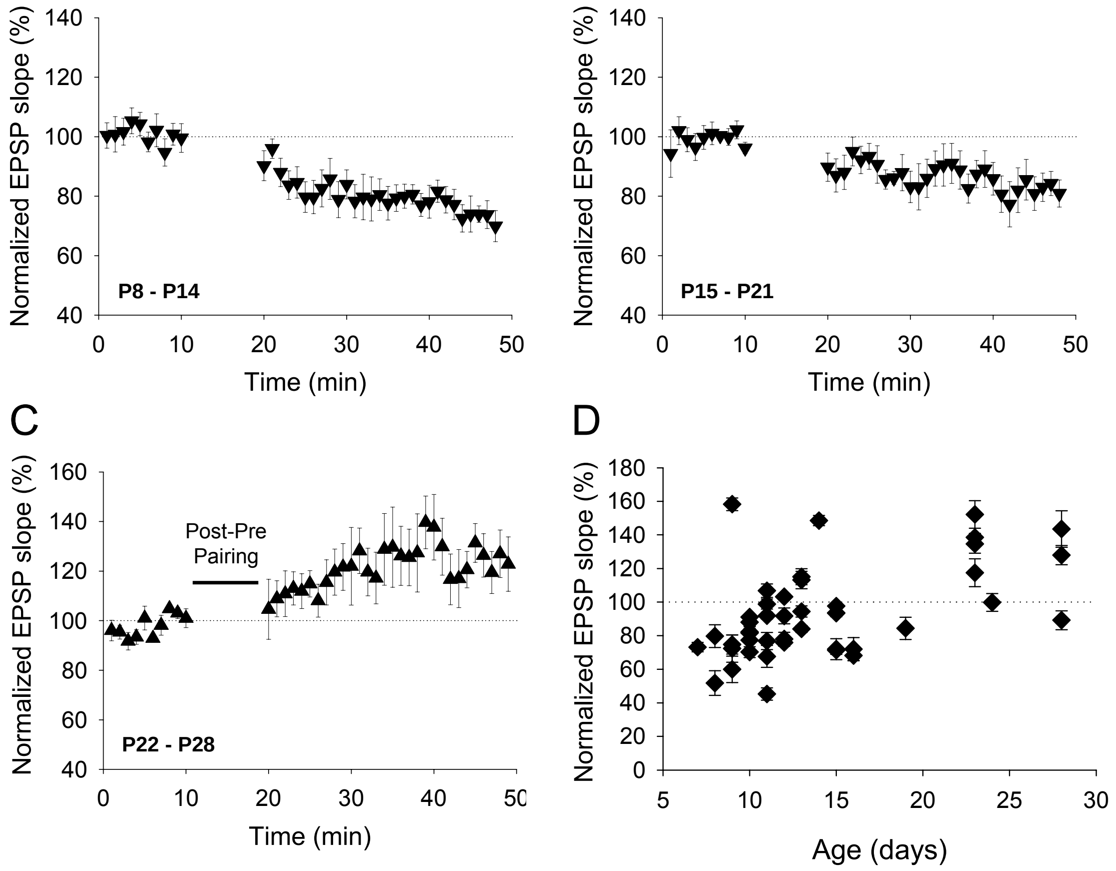

Figure S6. Developmental profile of t-LTD at CA3-CA1 synapses of the mouse hippocampus. Synaptic efficacy was monitored over time following post-before-pre single-spike pairing protocol in (A) P8-P14, n = 28; (B) P15-P21, n = 9; and (C) P22-P28 mice, n = 8. Note a switch from t-LTD to t-LTP after three weeks of development. (D) Summary of results. Error bars are S.E.M.
